# Engineering Nanoplatforms for Alzheimer's Disease Detection via Biomolecular Corona Proteomic and Lipidomic Profiling

**DOI:** 10.1002/adhm.71441

**Published:** 2026-07-11

**Authors:** Antonietta Greco, Roberto Baretta, Andrea Di Fonzo, Andrea Armirotti, Stefano Cinti, Mara Mirasoli, Marco Frasconi, Claudia Corbo

**Affiliations:** ^1^ School of Medicine and Surgery Nanomedicine Center Nanomib University of Milano Bicocca Vedano al Lambro MB Italy; ^2^ Department of Chemical Sciences University of Padova Padova Italy; ^3^ Analytical Chemistry Lab Istituto Italiano di Tecnologia Genoa Italy; ^4^ Department of Pharmacy University of Naples “Federico II” Naples Italy; ^5^ Sbarro Institute for Cancer Research and Molecular Medicine Center for Biotechnology College of Science and Technology Temple University Philadelphia Pennsylvania USA; ^6^ Bioelectronics Task Force at University of Naples Federico II Naples Italy; ^7^ Department of Chemistry “G. Ciamician” University of Bologna Bologna Italy; ^8^ IRCCS Istituto Ortopedico Galeazzi Milan Italy

**Keywords:** Alzheimer's disease, biomolecular corona, diagnosis, mass spectrometry, nanoparticles

## Abstract

The escalating global prevalence of Alzheimer's disease (AD) requires the development of sensitive, non‐invasive diagnostic strategies capable of detecting pathological changes previous the clinical onset. By exploiting the spontaneous formation of the biomolecular corona (BC) around nanoparticles (NPs), it's possible to capture a rich, disease‐specific fingerprint from the plasma that reflects systemic alterations often invisible to traditional diagnostic assays. In the present study, we demonstrate the efficacy of an innovative nanotechnological platform utilizing a synergistic dual‐silica NPs system (comprising amino‐ and sulfonate‐functionalized surfaces) integrated with proteomic and lipidomic profiling of the NP‐associated BC. Our results revealed a significant enrichment of ribosomal machinery in the BC of AD patients, contrasted by a simultaneous decrease of glycolytic enzymes and mitochondrial respiratory components. This metabolic impairment is further corroborated by the lipidomic profile, which shows a reduction in short‐chain acyl‐carnitines (C2, C3, C5) and essential membrane phospholipids. Additionally, the observed loss of structural proteins, such as Cofilin‐1 and Vinculin, contributes to a comprehensive molecular fingerprint unique to the AD phenotype.

## Graphical abstract

Engineered nanoparticles capture disease‐specific biomolecular coronas that uncover hidden molecular features of Alzheimer's disease. Combined proteomic and lipidomic analyses reveal a characteristic shift in ribosomal machinery and energy metabolism, generating a multiomic fingerprint that supports accurate disease detection and opens new opportunities for monitoring disease progression.

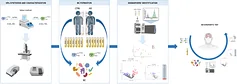

## 1 Introduction

Alzheimer's disease (AD), a common form of dementia, impacts approximately 10% of people over the age of 65, and its incidence is steadily rising worldwide [1, 2, 3]. This progressive neurodegenerative condition significantly impairs cognitive abilities, particularly memory and behavior [3, 4]. According to AD International website, the global number of individuals with dementia was over 50 million in 2020, a number projected to reach 82 million by 2030, and 152 million by 2050 (https://www.alzint.org/). Despite ongoing research, current diagnostic procedures for AD remain invasive and expensive, while the trend underscores the urgent need for advanced solutions such as nanomedicine‐based approaches to improve diagnosis, targeted drug delivery, neuroprotection, and therapeutic efficacy in the management of these diseases [1, 5, 6, 7]. Increasing evidence suggests that the pathological processes underlying AD begin decades before clinical symptoms appear, highlighting the unmet need for sensitive and accessible early diagnostic tools [1, 8]. At present, diagnosing AD is mostly based on medical history, cognitive function, the detection of biomarkers (Aβ42, tau, phosphorylated tau, and neurofilament light) in the cerebrospinal fluid (CSF), and neuroimaging such as positron emission tomography (PET) and magnetic resonance imaging (MRI) [9, 10].

However, since no single analysis reliably identifies the disease, multiple tests are often required, compounding the burden on patients. The development of a more accurate, non‐invasive diagnostic method remains a critical goal in the field [1, 11]. One promising approach involves analyzing blood‐based biomarkers [10]. Unlike CSF collection, blood draws are minimally invasive and can be performed repeatedly, facilitating both diagnosis and longitudinal monitoring [1, 9, 10, 12]. In recent years, our group and others have explored the use of nanoparticles (NPs) in biological fluids to uncover disease‐specific molecular patterns [1, 12, 13, 14, 15, 16]. When introduced into plasma, NPs quickly bind with proteins and other biomolecules to form a dynamic layer known as the biomolecular corona (BC) [17, 18, 19, 20, 21]. This BC changes the biological identity of the NPs and influences its interactions within biological systems [22]. The composition of the BC is shaped by the surrounding biomolecules, which can vary significantly with disease [23]. As a result, the BC may serve as an indirect biosensor that reflects disease‐specific alterations in the plasma biomolecules. This opens up the potential for using personalized BC signatures to detect subtle biomolecular changes that cannot be observed with conventional assays [12, 17, 22]. Indeed, prior recent studies have leveraged this concept for detecting cancers, cardiovascular diseases, neurodegenerative diseases, and viral infections using techniques to investigate the BC such as dynamic light scattering (DLS), general imaging analysis, 1D‐SDS‐PAGE, and mass spectrometry (MS) [1, 12, 13, 14, 24, 25, 26, 27].

In 2021, Hadjidemetriou et al. demonstrated the potential of the BC for AD‐related biomarker discovery. By intravenously administering lipid NPs into an AD mouse model and recovering them from circulation at multiple disease stages, the authors showed that the composition of the in vivo BCs reflected longitudinal changes in the blood proteome associated with AD pathology [24]. In the same year, Corbo et al. developed a platform based on six NPs with diverse surface functionalizations designed to detect AD‐specific protein signatures for early diagnosis. Through liquid chromatography‐tandem MS (LC‐MS/MS) analysis of the NP‐associated coronas combined with machine learning approaches, the authors successfully discriminated between AD and control samples, achieving an overall diagnostic accuracy of approximately 90% [1]. Collectively, these studies provided the first proof‐of‐concept that NP‐enabled enrichment of circulating proteomic fingerprints can reveal subtle, patho‐physiologically relevant biomarkers for the early detection of AD.

More recently, the predominant trend in AD nano‐diagnostics has shifted toward antibody‐functionalized NPs targeting a single pre‐selected biomarker such as Aβ or tau [28]. In parallel, corona‐engineering strategies have been developed to deepen plasma proteome coverage by modulating a single NP with panels of small molecules, though without addressing disease‐specific stratification or the lipidomic layer of the BC [29].

Within this evolving landscape, an untargeted, multiplexed strategy capable of capturing both protein‐ and lipid‐level alterations directly relevant to AD pathophysiology, while remaining compatible with rapid clinical translation, has not yet been addressed.

The present work aims to advance NPs‐based AD diagnostics toward a more clinically scalable sensing strategy. Specifically, we sought to determine whether the diagnostic performance previously achieved using a six‐NPs platform could be preserved through a substantially simplified system based on the combined use of two NPs with distinct surface functionalization. Beyond proteomic profiling, we also characterize the lipid component of the BC to capture a broader disease‐associated molecular signature. The proposed nanoplatform consists of two types of silica (Si) NPs of comparable size, each bearing a single surface chemistry, either amino (‐NH_3_^+^) or sulfonate (‐SO_3_^−^) groups, which are mixed prior to plasma exposure for BC formation. This reductionist, yet synergistic design, is intended to enhance robustness, reproducibility, and scalability, key requirements for the future development of NP‐based diagnostic sensors suitable for clinical translation. From a physicochemical perspective, the use of amino‐ and sulfonate‐functionalized NPs is expected to promote complementary biomolecular adsorption driven primarily by electrostatic interactions, hydrogen bonding, and secondary hydrophobic effects. The term “ammonio” designates the protonated, cationic form of the grafted primary amino group (‐NH_2_ → ‐NH_3_^+^); aminopropyl‐functionalized silica NPs have been reported to reach isoelectric points up to ≈9.2 depending on amino‐group density, indicating that this surface group remains substantially protonated under the near‐neutral pH conditions (≈7.4) used for BC formation, accounting for its positive charge throughout this study [30]. Positively charged ammonio (‐NH_3_^+^) surface groups preferentially attract negatively charged biomolecules, including acidic plasma proteins enriched in aspartate and glutamate residues, as well as proteins with low isoelectric points such as albumin, apolipoproteins, and several inflammatory or complement‐related proteins. In addition, amino‐functionalized surfaces may favor interactions with negatively charged phospholipids, including phosphatidylserine and phosphatidylinositol species, which are known to be altered in neurodegenerative conditions [1, 12, 31, 32, 33]. On the other hand, sulfonate (‐SO_3_^−^) groups promotes the adsorption of positively charged or basic proteins, including those enriched in lysine and arginine residues, such as histones, coagulation factors, immunoglobulin fragments, and certain acute‐phase proteins. Sulfonate surfaces may also preferentially interact with lipid‐associated proteins and zwitterionic lipid species through a combination of electrostatic screening and hydrogen bonding [12, 31, 32, 33, 34].

Taken together, simultaneous exposure of plasma to amino‐ and sulfonate‐functionalized NPs is expected to broaden the biomolecular capture spectrum, enriching partially overlapping yet distinct subsets of proteins and lipids. This synergistic adsorption provides a mechanistic rationale for generating richer and more discriminatory BC signatures, potentially increasing sensitivity to subtle, disease‐associated alterations in the plasma proteome and lipidome characteristic of AD.

## 2 Experimental Details

### 2.1 Materials

Tetraethyl orthosilicate (TEOS, 98%), (3‐mercaptopropyl)trimethoxysilane (MPTMS, 95%), (3‐aminopropyl)trimethoxysilane (APTMS, 97%), 5,5′‐dithio‐bis‐(2‐nitrobenzoic acid) (DTNB, 99%), ethylenediaminetetraacetic acid disodium salt dihydrate (EDTA, 99%), sodium phosphate monobasic dihydrate (*>* 99.0%), sodium phosphate dibasic dihydrate (*>* 99.0%), ninhydrin, ammonia solution (NH_3_, 32%), nitric acid (HNO_3_, 65%), ethanol (> 99.8%) were all purchased from Sigma Aldrich Co (St. Louis, MO).

Human plasma from AD patients and healthy (CTRL) individuals was purchased from Precision for medicine (Bethesda, MD, USA). All the information about the donors are collected in Table S2. Bradford, Laemmli buffer 4X, and 4–20% Mini‐PROTEAN TGX Pre‐cast Gels were obtained by Bio‐Rad Laboratories, Hercules, CA. Coomassie Brilliant Blue, urea, and ammonium bicarbonate were supplied from ThermoFisher Scientific (Fair Lawn, NJ, USA). All the materials used for the mass spectrometry analysis were supplied by ThermoFisher Scientific.

### 2.2 Nanoparticles Synthesis and Characterization

Silica bare NPs were synthesized using the Stöber method [35]. NPs were functionalized with APTMS to yield Si‐NH_3_^+^ NPs, or with MPTMS, followed by oxidation with HNO_3_, to obtain Si‐SO_3_^−^ NPs [36].

#### 2.2.1 Functionalization of Silica Nanoparticles with Amino Groups (Si–NH_3_^+^ NPs)

In 2 mL ethanol, (3‐aminopropyl)trimethoxysilane (APTMS, 2.4 µL) was added under magnetic stirring, followed by the addition of 6.7 µL of ammonia solution (1.48 m). The resulting mixture was added dropwise to a 200 µL DI water suspension of 10 mg Si NPs, and incubated for 5 hours under vigorous magnetic stirring at 75°C. After cooling, the suspension was centrifuged (14000 rpm, 5 min), and a white solid collected. The obtained solid was purified by six times redispersion in 4 mL of DI water/ethanol solution followed by centrifugation and supernatant removal. The product was then dried in vacuo at room temperature to obtain amino‐functionalized Si NPs (Si–NH_3_^+^ NPs, 9.9 mg) as a white solid [37].

#### 2.2.2 Functionalization of Silica Nanoparticles with Sulfonic Acid Groups (Si‐SO_3_^–^ NPs)

In 4 mL ethanol, (3‐mercaptopropyl)trimethoxysilane (MPTMS, 4.0 µL) was added under magnetic stirring, followed by the addition of 13.4 µL of ammonia solution (1.48 m). The resulting mixture was added dropwise to a 400 µL DI water suspension of 20 mg Si bare NPs, and incubated for 5 hours under vigorous magnetic stirring at 75°C. After cooling, the suspension was centrifuged, and a white solid collected. The obtained solid was purified by six times redispersion in 4 mL of DI water followed by centrifugation and supernatant removal. The product was then dried in vacuo at room temperature to obtain thiol‐functionalized Si NPs (Si–SH NPs, 19.8 mg) as a white solid. 9 mg of Si–SH NPs were dispersed in 1 mL of DI water and the resulting suspension was incubated with 1 mL of nitric acid (final concentration 7 m) under vigorous magnetic stirring for 5 hours at room temperature. The resulting suspension was centrifuged, followed by supernatant removal and collection of a white precipitate. The obtained solid was purified by six times redispersion in 4 mL of DI water followed by centrifugation and supernatant removal. The product was then dried in vacuo at room temperature to obtain sulfonic acid‐functionalized Si NPs (Si–SO_3–_ NPs, 7.1 mg) as a white solid [36].

#### 2.2.3 Quantification of Amino Groups on Si–NH_3_^+^NPs

A total of 3.3 mg of Si‐NH3^+^ NPs were dispersed in 300 µL DI water, followed by addition of 300 µL of ninhydrin 19.6 mM ethanol solution. The resulting mixture was incubated for 15 min under vigorous magnetic stirring at 95°C. After cooling to 25°C, the mixture was diluted to 2.0 mL by addition of 60% ethanol solution, followed by centrifugation (14 000 rpm, 5 min). The supernatant was collected and UV–vis spectrum (Agilent Cary 60 spectrophotometer) was recorded at 25°C using a quartz cuvette (1 cm path length). The absorbance at 570 nm was correlated to the concentration of amino groups through a calibration curve obtained from solutions of (3‐aminopropyl)trimethoxysilane (APTMS), ranging from 150 to 600 µm, in 60% ethanol [37].

#### 2.2.4 Quantification of Thiol Groups on Si‐SH NPs and Si‐SO_3_^–^ NPs

A volume of 100 µL of a 6.7 mg mL^−1^ Si‐SH NPs suspension in DI water was added to 890 µL solution comprising phosphate buffered saline (PBS) 0.1 m (pH 8.0), in the presence of 1 mm ethylenediaminetetraacetic acid disodium salt dihydrate (EDTA), and followed by addition of 10 µL of 5,5′‐dithio‐bis‐(2‐nitrobenzoic acid) (DTNB) 2.84 mm solution in PBS 0.1 m, pH 8.0, in the presence of 1 mm EDTA. The resulting mixture (final concentrations: 0.66 mg mL^−1^ of Si‐SH NPs, 1 mm EDTA, and 28.1 µm DTNB) was incubated at 25°C under vigorous magnetic stirring for 15 min, followed by centrifugation (14 000 rpm, 5 min). The supernatant was collected and UV–vis spectrum (Agilent Cary 60 spectrophotometer) recorded at 25°C using a quartz cuvette (1 cm path length). Analogue procedure was employed for evaluation of Si‐SO_3–_ NPs, briefly, by addition of 100 µL of a 6.7 mg mL^−1^ Si‐SO_3–_ NPs suspension to 890 µL of PBS 0.1 m solution, in the presence of EDTA and DTNB (final concentrations: 0.66 mg mL^−1^ of Si‐SO_3–_ NPs, 1 mm EDTA, and 28.1 µm DTNB), followed by 15 min incubation, centrifugation, supernatant collection and recording of UV–vis spectrum at 25°C. The absorbances at 411 nm were correlated to the concentration of thiol groups through a calibration curve obtained from solutions of (3‐mercaptopropyl)trimethoxysilane (MPTMS), ranging from 10 to 250 µm, in PBS 0.1 m, pH 8.0, in the presence of 1 mm EDTA [38].

#### 2.2.5 Nanoparticles Physico‐Chemical and Morphological Characterization

The mean diameter, polydispersity index (PDI), and superficial charge of NPs were measured at room temperature by dynamic light scattering (DLS) using the Brookhaven Instruments Corporation, Holtsville, NY, USA, equipped with a ZetaPALS device. All the analyses were performed at least in triplicate for each formulation and results were reported as mean ± Standard Deviation (S.D.).

The morphology and microstructure of the NPs were characterized by transmission electron microscopy (TEM) using a TEM JEOL JEM F200 operated at 200 kV. A carbon supported copper grids, 400 mesh size, were used for preparation of the sample.

### 2.3 Biomolecular Corona Formation

Human plasmas from AD patients and healthy control (CTRL) individuals were collected in K_2_EDTA tubes and centrifuged at 500 × *g* for 20 min (room temperature). The plasma supernatant was transferred to a new tube without disturbing the cell layer. To increase the plasma purity, a second centrifugation was carried out at 1000 × *g* for 5–10 min (room temperature). Plasma was aliquoted and frozen at ‐80°C for long‐term storage. To obtain BC from AD (*n* = 7) and CTRL plasma samples (*n* = 7), a total amount of 0.5 mg (1:1 w:w) of Si–SO_3_^−^ and Si–NH_3_^+^ was incubated at 37°C for 1 h under agitation with 200 µL of human plasma samples diluted with 200 µL of DI water (final plasma concentration 50% v/v). Following the incubation, the BC‐coated NPs were separated by centrifugation at 14 000 × *g* at 4°C for 30 min and, to remove the soft BC, three independent washing with 4°C cold PBS were carried out. To verify that our washing procedure effectively removes unbound proteins and non‐associated aggregates prior to corona analysis, as part of our standard workflow, we routinely performed a Bradford protein assay on the wash supernatants and increase the number of washing steps if any residual protein is detected. In the experiments reported here, the protein assay performed on the washing buffer collected after the final wash showed a protein concentration below the detection limit (effectively zero), indicating that no free proteins remained following centrifugation and washing. The BC‐NPs complexes in the pellet were resuspended in a denaturing buffer (8 m urea and 50 mm ammonium bicarbonate) for 1D gel electrophoresis analysis. The proteins concentration within the BC was measured by Bradford assay following the manufacturer's protocol instructions and by using albumin for the standard curve.

### 2.4 1D‐SDS‐PAGE

An equal volume of Laemmli buffer 4X (Bio‐Rad Laboratories, Hercules, CA) was added to BC samples previously dissolved in 8 m urea and 50 mm ammonium bicarbonate. After incubation at 90°C for 5 min, proteins were separated on 4–20% Mini‐PROTEAN TGX Precast Gels (Bio‐Rad Laboratories, Hercules, CA) under constant voltage (120 V, 1 hour). The resulting protein bands were stained overnight with Coomassie Brilliant Blue (ThermoFisher Scientific, Fair Lawn, NJ, USA) and extensively washed with ultrapure water. Gel images were captured using the Amersham Imager 600 (GE Healthcare) and analyzed with ImageJ software (imagej.nih.gov/ij/).

### 2.5 Protein Identification and Quantification by Mass Spectrometry

#### 2.5.1 Protein Digestion

To prepare the BC‐derived proteins for MS analysis, the isolated pellets were first resuspended in a reduction/alkylation buffer consisting of 10 mm tris(2‐carboxyethyl)phosphine (TCEP) and 30 mm chloroacetamide in 50 mm tetraethylammonium bromide (TEAB). The samples were incubated at 95°C for 15 min in dark conditions to ensure complete denaturation. Following incubation and cooling to room temperature, the proteins were processed using the single‐pot solid‐phase‐enhanced sample preparation (SP3) protocol [39], an approach selected for its high efficiency and robustness in handling small‐volume clinical samples. The resulting peptide concentration was determined using a fluorometric peptide assay, and a standardized amount of 300 ng of peptides per sample were used for each proteomic analysis.

#### 2.5.2 Mass Spectrometry Data Acquisition and Analysis

The proteomics analysis was conducted on a Thermo Exploris 480 orbitrap system coupled with a Dionex Ultimate 3000 nano‐LC system. Peptides were first trapped and desalted before being loaded onto an Aurora C18 nanocolumn (75 mm x 250 mm, 1.6 µm particle size; Ion Opticks, Australia). The separation was achieved through a 1‐hour linear gradient of acetonitrile (ACN) (3% to 41%) in water, both supplemented with 0.1% formic acid, at a constant flow rate of 300 nL min^−1^. The total run time, including column reconditioning, was 90 min. Injection volume was set to 1 µL. MS data were acquired in positive Electrospray ionization (ESI) mode with a capillary voltage of 2.0 kV. To maximize the depth and reproducibility of the proteomic coverage, the instrument was operated in Data Independent Acquisition (DIA) mode. Survey scans were performed across a mass range of 400 to 1000 m/z at a resolution of 120 000, followed by MS/MS acquisition using 60 m/z transmission windows, each having a fixed 10 Da width. MS/MS spectra were acquired in higher‐energy collisional dissociation (HCD). All the collected MS/MS spectra were analyzed using Spectronaut (Version 18), by running a DirectDIA analysis against the reference Homo Sapiens FASTA database (Tax ID: 9606 reporting 51548 reviewed entries). Cysteine carbamidomethylation was selected as fixed modification; acetylation of protein N‐Term and methionine oxidation were selected as variable modifications. Positive protein identifications were retained at 1% false discovery rate (FDR) threshold and at least two peptides were used.

### 2.6 Lipids Identification and Quantification by Mass Spectrometry

Lipid extraction was performed by adding 1 mL of an isopropanol (IPA)–based protein precipitation solution containing ceramide 17 (Cer17) at a concentration of 0.7 µm as an internal standard. After extraction, samples were dried under vacuum and reconstituted in 100 µL of solvent, resulting in a tenfold increase in the final internal standard concentration (7 µm). The mixtures were incubated for 15 min at room temperature on an orbital shaker at 1000 rpm. Following incubation, the samples were subjected to sonication in an ice‐water bath for 10 min and subsequently centrifuged at 20 000 x *g* for 20 min at 4°C. The resulting supernatants were transferred to fresh tubes, while the remaining pellets were stored at ‐20°C for subsequent proteomic analysis. The lipid‐rich supernatants were dried under a nitrogen flow and stored at ‐20°C. Prior to analysis, the dried extracts were reconstituted in a 9:1 (v/v) mixture of methanol and chloroform. To monitor analytical consistency, quality control (QC) samples were prepared by pooling representative aliquots from each sample. Both randomized experimental samples and QCs were analyzed using a UHPLC Shimadzu ExionLC AE system coupled with a Sciex 7500+ quadrupole‐trap mass spectrometer (Sciex, Framingham, USA). The lipidomic analysis was conducted using an optimized Ultra‐high‐performance liquid chromatography (UHPLC) protocol. Separation was achieved on a CHS C18 column (2.1×50 mm, 1.7 µm particle size; waters, Milford, USA) with the column heater maintained at 55°C. The mobile phase system was composed of (A) 40:60 H_2_O/ACN (v/v) and (B) 90:10 IPA/ACN (v/v), both supplemented with 10 mm ammonium formate. All materials used were supplied by ThermoFisher Scientific. Chromatographic elution was performed at a constant flow rate of 0.450 mL/min according to the following gradient profile: the initial condition of 10% B was held for 1.00 minute, followed by a linear increase to 60% B at 4.00 min and 75% B at 8.00 min. The organic phase reached 100% B at 9.50 min and was maintained until 11.00 min to ensure complete elution. The system was then returned to 10% B at 11.10 min and re‐equilibrated until 13.00 min. Quantification of the global lipidomic profile was executed via multiple reaction monitoring (MRM) transitions (Table 1).

**TABLE 1:** MRM transitions used for the MS/MS quantification of the global lipidomic profiling.

Time (min)	Flow rate (mL min^−1^)	% B
Initial	0.450	10%
1.00	0.450	10%
4.00	0.450	60%
8.00	0.450	75%
9.50	0.450	100%
11.00	0.450	100%
11.10	0.450	10%
13.00	0.450	10%

#### 2.6.1 Mass Spectrometry Data Acquisition and Analysis

MS detection was performed utilizing a source temperature of 450°C, with the curtain gas set to 40 psi and the spray voltage maintained at 1800 V. Lipids were targeted and quantified via MRM; the specific transitions employed for the global lipidomic profiling are detailed in Table 1. Downstream data processing and functional interpretation were conducted using the following specialized computational platforms: **Perseus (version 1.6.15.0)**: used for the comprehensive statistical analysis and normalization of the high‐dimensional omics datasets [40]. **MetaboAnalyst 6.0**: employed as a unified web‐based platform for lipidomic data processing, metabolic pathway analysis, and results interpretation [41]. **DAVID (2021 Update)**: used for functional enrichment analysis and biological annotation to identify significant gene and protein clusters associated with the identified biomarkers [42, 43].

### 2.7 Statistical Analysis

All results are presented as mean value ± S.D. Comparisons between AD and CTRL groups (e.g., densitometric protein intensity, Figure 3D) were performed using an unpaired, Student's t‐test. Receiver operating characteristic (ROC) curve and area under the curve (AUC) analyses were performed to evaluate the diagnostic performance of the platform, with sensitivity and specificity reported at the optimal cutoff. These statistical evaluations were performed in GraphPad Prism version 8.3.0 (GraphPad Software Inc., La Jolla, CA, USA). For proteomic data, normalization, principal component analysis (PCA), and differential abundance analysis (volcano plots, Log2 fold change vs. ‐Log10 *p*‐value) were performed in Perseus (version 1.6.15.0), with statistical significance defined at an adjusted *p*‐value < 0.05. For lipidomic data, statistical processing and pathway analysis were performed in MetaboAnalyst 6.0, with significance defined at *p* < 0.05. Functional enrichment and biological annotation of significant protein/lipid clusters were performed using DAVID (2021 Update) [40, 41, 42, 43].

All experiments were conducted with n = 7 biological replicates per group (AD and CTRL) unless otherwise specified.

## 3 Results and Discussion

**FIGURE 1:**
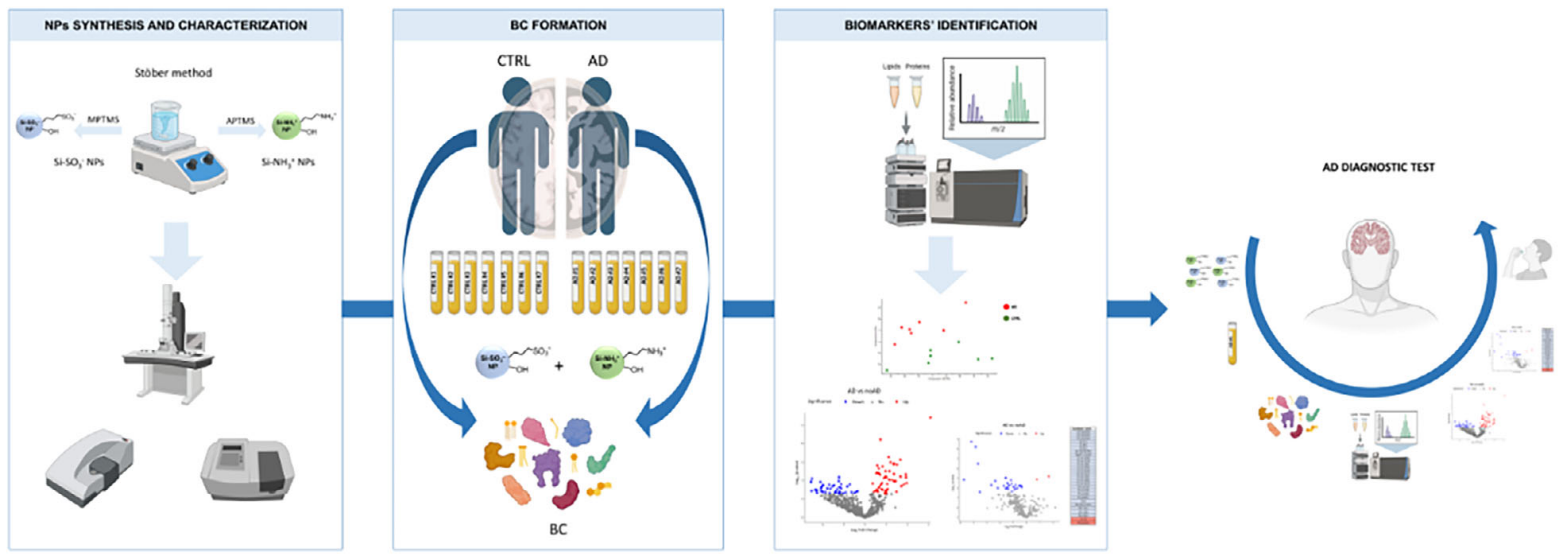
Schematic workflow of the NP‐BC‐based diagnostic platform for AD. The experimental pipeline comprises three integrated phases: 1) NP synthesis and characterization: Si bare NPs functionalized with amino (Si‐NH_3_^+^) or sulfonate (Si‐SO_3_^−^) groups were synthesized and characterized. 2) Biomolecular corona (BC) formation: Plasma samples from AD patients and healthy controls (CTRL) were incubated with a mixture of the two NP types, allowing the formation of a BC that concentrates disease‐associated biomarkers. 3) Biomarker identification: The resulting BCs were isolated and analyzed, and multivariate statistical analyses were applied to identify unique proteomic and lipidomic signatures. Integrating these phases supports the development of a reliable NP‐BC‐based diagnostic tool for AD. Created in https://BioRender.com.

An overview of the experimental workflow is illustrated in Figure 1. Plasma from AD patients and healthy individuals (CTRL) was incubated with a tailored cocktail of combined aminated and sulfonated Si NPs (Si‐NH_3_^+^ and Si‐SO_3_^−^), synthesized in our laboratory. This step was followed by isolation of the BCs, purification, characterization, and proteomics and lipidomics analysis to reveal compositional discriminatory signatures further validated by statistical tests.

**FIGURE 2:**
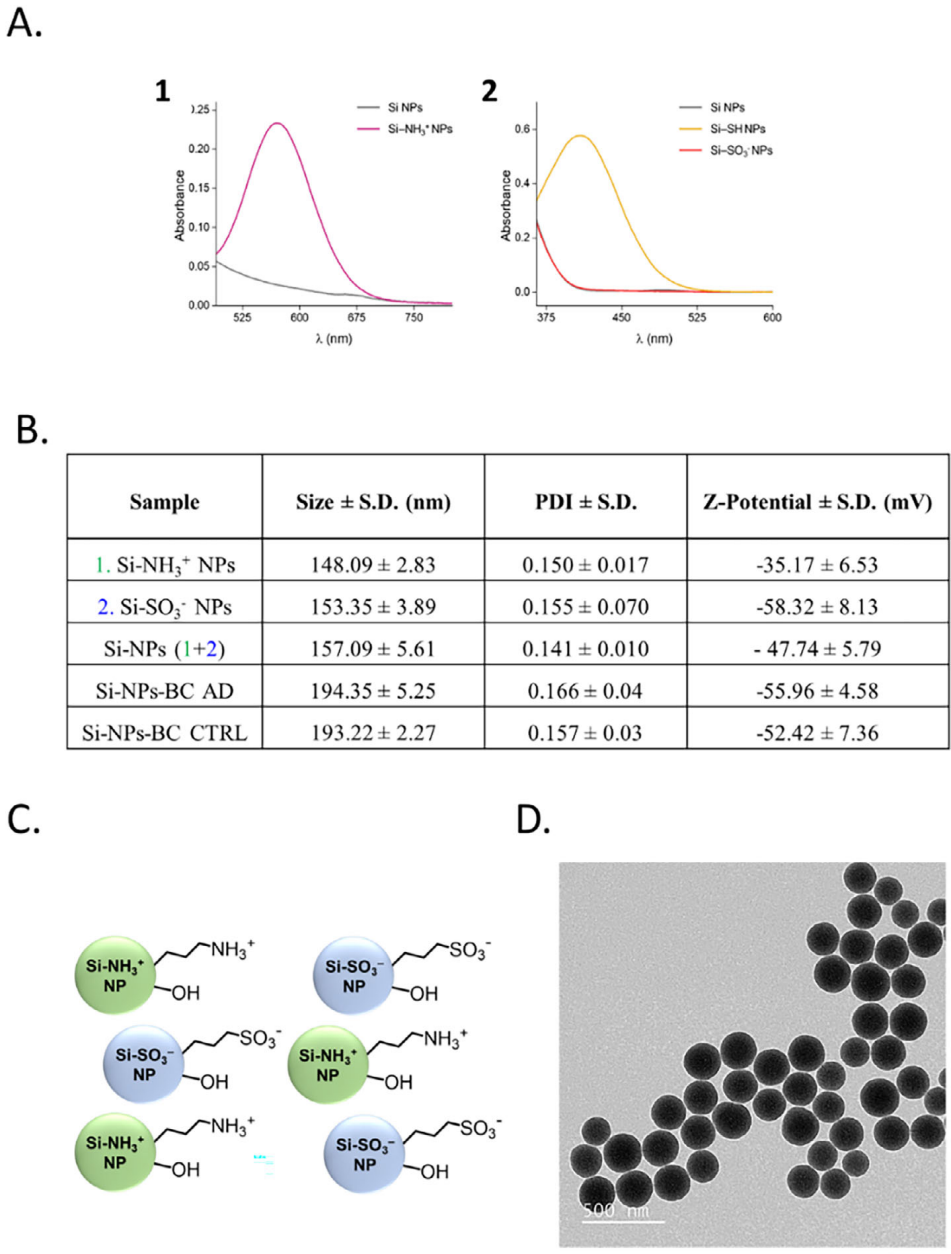
NPs characterization. A. UV–Vis absorption spectra for the quantification of surface functional groups on NPs. 1. UV–Vis spectra of the supernatant solution obtained from Si–NH_3_^+^ NPs (purple curve) and Si NPs (gray curve) after treatment with ninhydrin. 2. UV–Vis spectra of supernatant solution from Si–SH NPs (orange curve), Si‐SO_3–_ NPs (red curve) and Si NPs (gray curve) following reaction with Ellman's reagent (DTNB). B. Size, homogeneity, and charge surface characterization of Si‐NH_3_^+^, Si‐SO_3_^−^, Si‐NPs (Si‐NH_3_^+^ + Si‐SO_3_^−^_1:1) bare and after incubation with AD and healthy human plasma. Results represent the mean ± S.D. of 7 independent batches (*n* = 7). C. Representation of the Si‐NPs cocktail (Si‐NH_3_^+^ and Si‐SO_3_^−^ 1:1) used in this study. D. TEM image: morphological and structural characterization of Si‐NPs cocktail (Si‐NH_3_^+^ and Si‐SO_3_^−^ 1:1) (scale bar = 500 nm).

Si bare NPs were grafted with APTMS to obtain Si‐NH_3_^+^ NPs, or with MPTMS followed by oxidation of the surface ‐SH groups with HNO_3_ to yield Si–SO_3_^−^ NPs, and the functionalization was confirmed by UV‐Vis spectroscopy (Figure 2A). The functionalization protocols were designed to achieve partial surface coverage with the ‐NH_3_^+^ or ‐SO_3_^−^ groups, 73% and 53% respectively, yielding a mixed surface chemistry composed of well‐spaced ‐NH_3_^+^ or ‐SO_3_^−^ groups, interspersed with residual silanol (Si‐OH) moieties, mimicking the chemical heterogeneity of biological interfaces. The surface coverage of –NH_3_^+^ functional groups on Si–NH_3_^+^ NPs was quantified using the ninhydrin assay (see Section “*Quantification of amino groups on Si–NH_3_^+^NPs*” for the detailed procedure) and compared to the reported surface silanol (–OH) concentration per gram of Si NPs (0.30 mmol g^−1^ for NPs of ≈130 nm diameter) [44]. Based on the absorbance measured at 570 nm for ninhydrin‐treated Si–NH_3_^+^ NPs (Figure 2A.1) and the corresponding calibration curve, a –NH_3_^+^ surface coverage of 0.22 mmol g^−1^ was determined, corresponding to 73 ± 4% functionalization of surface –OH groups. As a control, pristine Si NPs treated with ninhydrin showed no significant absorbance.

Functionalization of Si–SO_3_^−^ NPs was achieved through a two‐step procedure. In the first step, MPTMS was grafted on the Si NPs to introduce thiol (–SH) groups on the surface, yielding Si–SH NPs. The surface coverage of –SH groups was quantified using Ellman's assay (see Section “*Quantification of thiol groups on Si‐SH NPs and Si‐SO_3–_ NPs*” for the detailed procedure) and then compared to the surface coverage of –OH groups (0.30 mmol g^−1^). From the measured absorbance at 411 nm (Figure 2A.2) and the appropriate calibration curve, a surface coverage of 0.16 mmol g^−1^ was obtained, corresponding to the functionalization of 53 ± 5% of –OH groups on the NP surface. As a control, Si NPs treated with Ellman's reagent (DTNB) exhibited no significant absorbance. In the second step, surface –SH groups were oxidized to –SO_3_^−^ groups by addition of nitric acid, yielding Si–SO_3_^−^ NPs. The absence of a significant absorbance at 411 nm upon treatment of the resulting Si–SO_3_^−^ NPs with Ellman's reagent confirms the complete oxidation of thiol groups to sulfonate functionalities. The physicochemical characterization of both Si‐NH_3_^+^ and Si‐SO_3_^−^ NPs was completed by measuring the hydrodynamic diameter, polydispersity index (PDI), and Z‐potential of the NPs by DLS analysis (Figure 2B) individually and as mixture (1:1_w:w graphically represented in the Figure 2C), before and after the incubation with AD and CTRL plasmas. Prior to plasma exposure, both Si‐NH_3_^+^ NPs and Si‐SO_3_^−^ NPs resuspended in water exhibited high homogeneity, with diameters consistently ranging between 148.09 ± 2.83 and 153.35 ± 3.89 nm (Figure 2B), in agreement with TEM analysis of the NPs mixture (Figure 2D). TEM analysis was performed also individually on the pristine and functionalized Si NPs (Figure S1). Pristine Si NPs exhibited an average diameter of 150 ± 20 nm (Figure S1A). TEM images of Si–NH_3_^+^ NPs (Figure S1B) revealed an average diameter of 163 ± 20 nm, comparable to that observed for Si–SO_3_^−^ NPs (Figure S1C), which displayed an average diameter of 170 ± 18 nm. These results indicate that the functionalization reactions performed on Si NPs do not induce morphological changes in the NPs, providing only a controlled surface functionalization.

**FIGURE 3:**
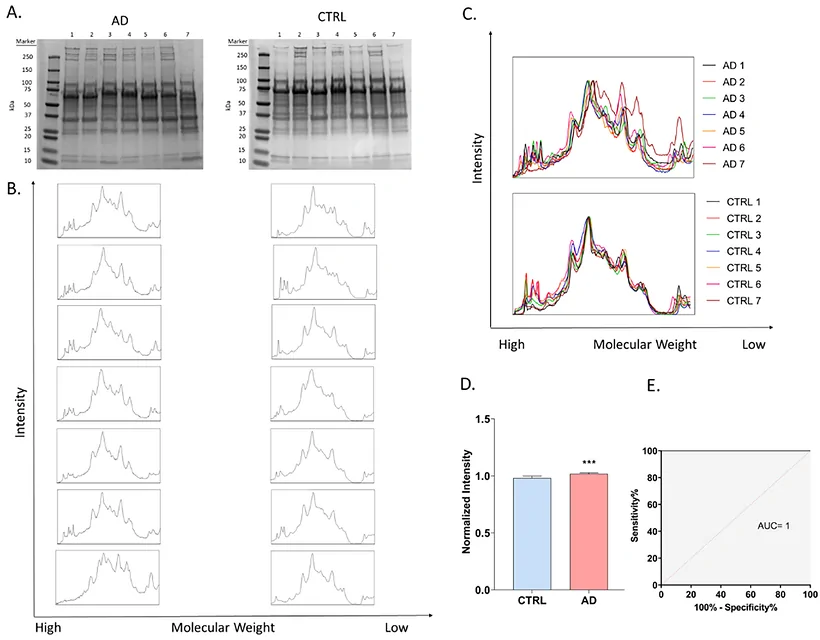
1D‐SDS‐PAGE gels and densitometric analysis of BC profiles. A. 1D‐SDS‐PAGE gels of protein profile derived from AD BC (*n* = 7) and CTRL BC (*n* = 7). B. Densitometric comparison between AD and CTRL protein profiles. C. Intra‐sample densitometric analysis comparison. D. Densitometric analysis of Si‐NPs’ BCs in AD vs healthy plasma. E. ROC curve and AUC analysis of Si‐NPs densitometric data to validate the performance of the diagnostic test. All the densitometric analysis were performed using ImageJ. Unpaired t‐tests determined statistical significance between groups. ***p ≤ 0.001.

The size distribution (157.09 ± 5.61 nm) and the PDI (0.141 ± 0.010) remained unchanged when the NPs were analyzed as a mixture demonstrating that no significant inter‐particle interference or aggregation occurred upon mixing. The physicochemical stability of the NPs mixture was a key prerequisite for our diagnostic approach. This demonstrates that the distinct NPs species did not interfere with each other, ensuring that the surface conjugated groups remained independently accessible, allowing us to fully exploit their specific surface chemical characteristics for the identification of AD diagnostic biomarkers. Following incubation in both AD and healthy human plasma, the NPs‐BC complexes showed a slight increase in mean size (194.35 ± 5.25 and 193.22 ± 2.27 nm, respectively) still maintaining a homogeneous distribution profile (PDI values of 0.166 ± 0.04 and 0.157 ± 0.03 nm, respectively) (Figure 2B). Consistent with previous reports, we observed an average diameter increase of approximately 30–40 nm, which indicates the formation of a BC layer with a thickness of roughly 15–20 nm [1, 12, 21, 45, 46]. The Z‐Potential characterization revealed that the surface charge of all investigated NPs was always negative (Figure 2B). In particular, Si‐NH_3_^+^ NPs presented a surface charge of ‐35.17 ± 6.53 mV. This value was significantly different (Student's t‐test, *p* < 0.05) from those measured for Si‐SO_3_^−^ (‐58.32 ± 8.13 mV) and for the Si NPs cocktail (Si‐NH_3_^+^ + Si‐SO_3_^−^), which exhibited a Z‐potential of −47.74 ± 5.79 mV, consistent with an intermediate charge arising from the combination of the two nanoparticle populations. When the combined Si‐NPs were incubated into both human AD and CTRL plasma, the charge became more negative, with Z‐potential reaching ‐55.96 ± 4.58 and ‐52.42 ± 7.36 mV, respectively. Such values are in line with literature for protein‐coated Si NPs [1, 12, 45]. Physicochemical characterization of the Si–NPs showed minor differences between AD and CTRL plasma, insufficient to act as a primary discriminator for disease detection. The results related to the size, PDI, and Z‐Potential of the Si‐NH_3_^+^ NPs and Si‐SO_3_^−^ NPs individually incubated with AD and CTRL plasma are reported in Table S1. Following incubation with either AD or CTRL plasma, statistically significant differences in Z‐potential were confirmed by pairwise comparisons among Si–NH_3_^+^ NPs, Si–SO_3_^−^ NPs, and the Si NP cocktail (Student's t‐test, *p* < 0.05) (Table S1). Next, we analyzed the proteomic profile of the BCs from both AD and CTRL plasma using 1D‐SDS‐PAGE, followed by Coomassie brilliant blue staining for band visualization (Figure 3A). Analysis of intra‐group variability among biological replicates of the same condition revealed highly reproducible protein profiles (Figure 3B), evidenced by the substantial overlap of densitometric intensity curves (Figure 3C), confirming that the BC formation process is robust across biological replicates. Moreover, comparative evaluation of the densitometric profiles of the AD and CTRL cohorts identified a stable set of proteins across all samples, with subtle differences in the band intensity in the molecular weight (MW) range between 20 and 50 kDa. These qualitative observations require further evaluation by MS, due to the limited resolution of the 1D gel electrophoresis.

To further characterize the differences between BCs formed upon incubation of Si‐NPs with CTRL (*n* = 7) and AD (*n* = 7) plasma, the average densitometric protein profiles across the full molecular weight range (10–250 kDa) were quantified using ImageJ (Figure 3D). This analysis revealed statistically significant differences in the BCs generated on the mixed Si‐NPs between healthy and AD samples. In line with these findings, the diagnostic performance of the platform was evaluated by ROC analysis and the corresponding AUC (Figure 3E), which confirmed the ability of the Si‐NPs system to discriminate between the two groups, supporting its potential relevance for clinical screening. At the optimal cutoff, the densitometric BC signature achieved 100% sensitivity and 100% specificity (AUC = 1.00). Given the limited cohort size (*n* = 7 per group), this result should be regarded as proof‐of‐concept and warrants confirmation in an independently recruited, larger validation cohort.

**FIGURE 4:**
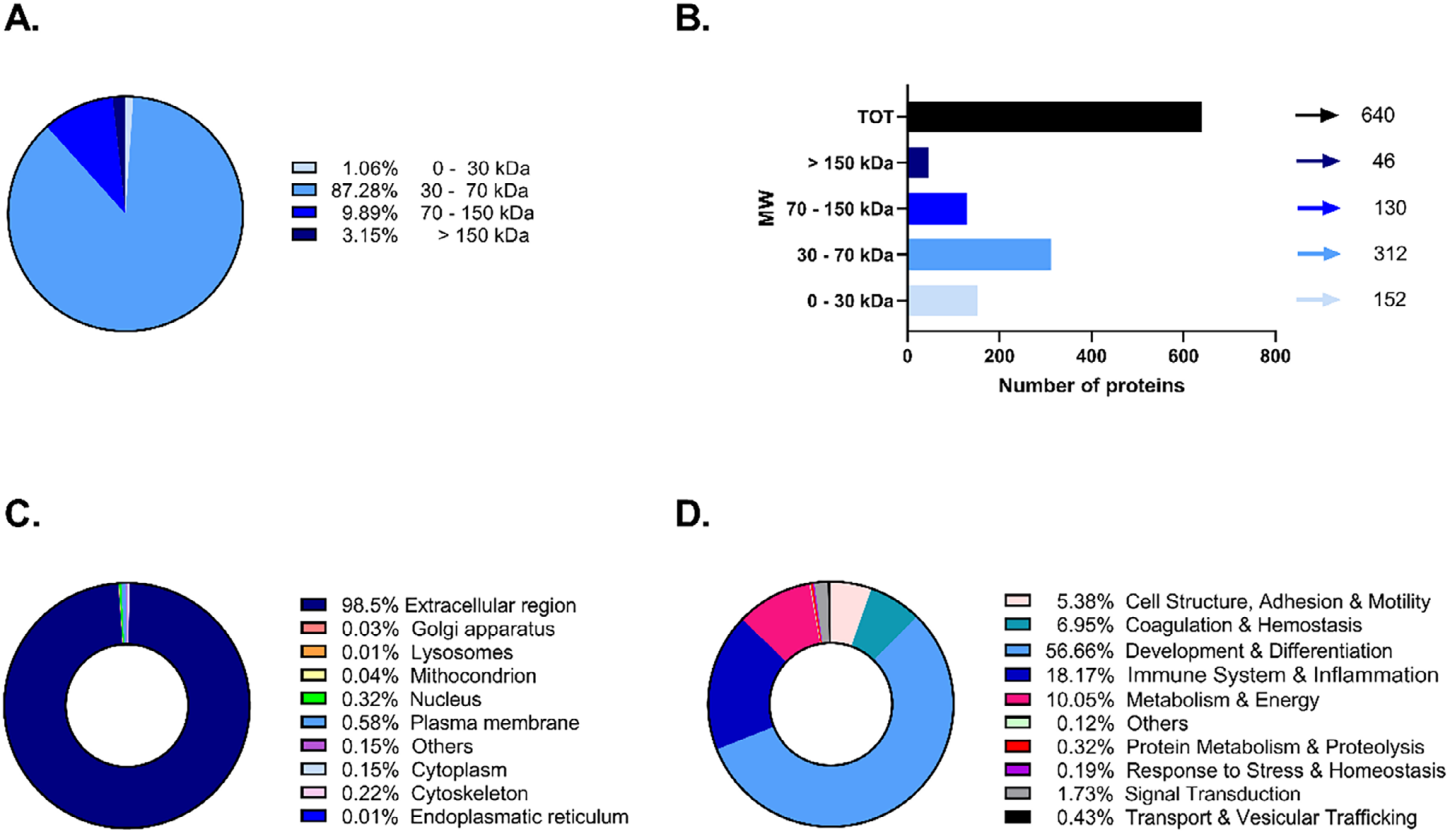
Features of proteins in the corona. A. Percentage distribution of proteins based on MW. B. Numeric protein distribution based on the MW. The average ratio of major protein cellular localization (C) and biological functions (D) clusters.

To accurately discriminate BC profiles of AD from controls and identify potential AD‐specific BC signature, we analyzed the samples by LC‐MS/MS. We performed a comprehensive proteomic analysis of the BC profiles of each group quantifying a total of 640 proteins, 87% of which with a MW in the range of 30–70 kDa (Figure 4A,B). As expected, proteins were mainly located in the extracellular compartment (98.5%) and they were primarily involved in development and differentiation biological processes (≈ 57%) (Figure 4C,D).

**FIGURE 5:**
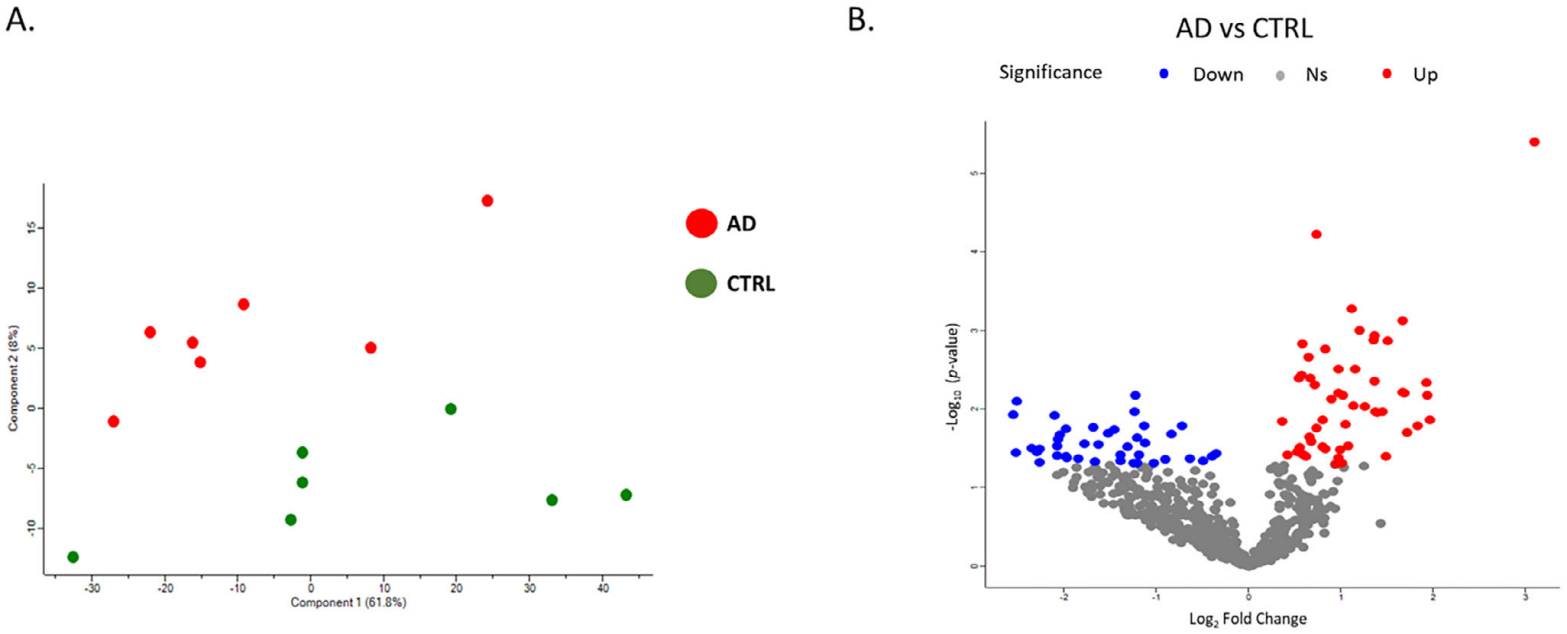
Proteomic analysis. A. PCA of the BC proteome. The graph illustrates the multivariate separation between AD patients (red) and healthy controls (green) based on LC‐MS/MS proteomic profiling. Component 1 and Component 2 account for 61.8% and 8% of the total variance, respectively. The distinct clustering along the PC1 axis demonstrates that the NPs‐integrated platform effectively captures a differential protein signature, providing a robust molecular basis for the discrimination of the AD phenotype from non‐pathological states. B. Differential expression analysis of the AD‐derived BC proteome. The volcano plot illustrates the protein abundance changes between AD and CTRL groups. Each point represents a unique protein identified via LC‐MS/MS. The x‐axis indicates the magnitude of change (Log_2_ fold change), while the y‐axis represents the statistical significance (‐Log_10_ (*p*‐value)). Proteins significantly more abundant in the AD cohort are shown in red, while less abundant proteins are shown in blue. Gray dots represent proteins that did not meet the statistical significance threshold (adjusted *p*‐value > 0.05). The plot highlights a robust proteomic signature in AD samples, characterized by a significant enrichment of ribosomal machinery components.

To explore proteomic variance, PCA was applied to normalized protein samples, revealing group‐related clustering patterns (Figure 5A). The analysis indicated a separation between AD and healthy participants forming two distinct clusters, thus indicating differential BC composition due to AD condition, suggesting the presence of proteins relevant for biomarker discovery.

Consistent with the variability patterns observed in the PCA analysis, differential expression analysis was performed to further characterize proteomic differences between groups. The results are visualized in a volcano plot (Figure 5B), which highlights proteins showing differential abundance between AD and healthy samples. This analysis revealed a distinct enrichment of specific proteins in the AD group, suggesting an altered plasma environment that may influence protein recruitment at the NPs’ surface. Proteins displaying relative increased and decreased abundance in AD are reported in Tables 2 and 3, respectively.

**TABLE 2:** AD vs CTRL: proteins with increased abundance.

Proteins with increased abundance				
Entry name	Gene name	Protein name	Log10 *p*‐value	Log2(FC)
CFAD_HUMAN	CFD	Complement factor D	5.405770734	3.10150433
IBP2_HUMAN	IGFBP2	Insulin‐like growth factor‐binding protein 2	1.866825552	1.96115971
FHR2_HUMAN	CFHR2	Complement factor H‐related protein 2	2.184240249	1.93159717
IBP6_HUMAN	IGFBP6	Insulin‐like growth factor‐binding protein 6	2.341836173	1.92420932
IPSP_HUMAN	SERPINA5	Plasma serine protease inhibitor	1.78792794	1.83104828
FA11_HUMAN	F11	Coagulation factor XI	1.710925967	1.71880477
KLKB1_HUMAN	KLKB1	Plasma kallikrein	2.210145481	1.687553
IBP4_HUMAN	IGFBP4	Insulin‐like growth factor‐binding protein 4	2.222254571	1.66864858
RNAS4_HUMAN	RNASE4	Ribonuclease 4	3.127679434	1.66770635
CD14_HUMAN	CD14	Monocyte differentiation antigen CD14	2.870499556	1.50599412
LV949_HUMAN	IGLV9‐49	Immunoglobulin lambda variable 9–49	1.403773959	1.48359939
TETN_HUMAN	CLEC3B	Tetranectin	1.976635243	1.4458204
KCRB_HUMAN	CKB	Creatine kinase B‐type	1.960064869	1.38952902
TBB5_HUMAN	TUBB	Tubulin beta chain	1.967412365	1.374947
RL18_HUMAN	RPL18	60S ribosomal protein L18	2.362588891	1.3642119
CCN5_HUMAN	CCN5	CCN family member 5	2.938965268	1.36417021
RL23_HUMAN	RPL23	60S ribosomal protein L23	2.885290505	1.34977538
A1AG2_HUMAN	ORM2	Alpha‐1‐acid glycoprotein 2	2.034698759	1.25396197
FCN3_HUMAN	FCN3	Ficolin‐3	3.007744426	1.20383126
ZA2G_HUMAN	AZGP1	Zinc‐alpha‐2‐glycoprotein	2.516987191	1.15382971
A1AG1_HUMAN	ORM1	Alpha‐1‐acid glycoprotein 1	2.045215028	1.1305403
ARF4_HUMAN	ARF4	ADP‐ribosylation factor 4	3.283769991	1.11644609
IC1_HUMAN	SERPING1	Plasma protease C1 inhibitor	1.535515502	1.07704435
HEMO_HUMAN	HPX	Hemopexin	1.810002348	1.04861123
TIF1B_HUMAN	TRIM28	Transcription intermediary factor 1‐beta	2.184556553	1.0216871
ANTR1_HUMAN	ANTXR1	Anthrax toxin receptor 1	1.314287528	1.00660501
RS25_HUMAN	RPS25	40S ribosomal protein S25	1.490786154	0.99049595
QSOX1_HUMAN	QSOX1	Sulfhydryl oxidase 1	2.209540068	0.974279
LCAT_HUMAN	LCAT	Phosphatidylcholine‐sterol acyltransferase	1.380499073	0.9719711
LEG3_HUMAN	LGALS3	Galectin‐3	2.512484655	0.97165081
CBPB2_HUMAN	CPB2	Carboxypeptidase B2	1.30829108	0.94864709
VDAC3_HUMAN	VDAC3	Voltage‐dependent anion‐selective channel protein 3	2.135664396	0.89890521
ASSY_HUMAN	ASS1	Argininosuccinate synthase	2.770295309	0.83162717
RL12_HUMAN	RPL12	60S ribosomal protein L12	1.496064925	0.82645212
RLA2_HUMAN	RPLP2	60S acidic ribosomal protein P2	1.865859836	0.80355835
A1BG_HUMAN	A1BG	Alpha‐1B‐glycoprotein	1.525808314	0.79662677
RL24_HUMAN	RPL24	60S ribosomal protein L24	1.765108677	0.73473195
ERP29_HUMAN	ERP29	Endoplasmic reticulum resident protein 29	4.236857498	0.73187583
H2AY_HUMAN	MACROH2A1	Core histone macro‐H2A.1	2.310158427	0.71637549
RS4X_HUMAN	RPS4X	40S ribosomal protein S4. X isoform	1.591183213	0.67436777
MPCP_HUMAN	SLC25A3	Phosphate carrier protein. Mitochondrial	2.397683124	0.6648597
HGFA_HUMAN	HGFAC	Hepatocyte growth factor activator	1.653447098	0.6551451
RS16_HUMAN	RPS16	40S ribosomal protein S16	2.661032719	0.64636203
IMDH2_HUMAN	IMPDH2	Inosine‐5'‐monophosphate dehydrogenase 2	1.403116801	0.62351036
SAMP_HUMAN	APCS	Serum amyloid P‐component	1.432698711	0.5830822
K2C7_HUMAN	KRT7	Keratin. type II cytoskeletal 7	2.839372008	0.57741955
BAG2_HUMAN	BAG2	BAG family molecular chaperone regulator 2	2.434840993	0.56791387
SSRP1_HUMAN	SSRP1	FACT complex subunit SSRP1	1.51167827	0.55457974
CFAH_HUMAN	CFH	Complement factor H	2.395636701	0.54455185
H15_HUMAN	H1‐5	Histone H1.5	1.462509861	0.52665602
KAIN_HUMAN	SERPINA4	Kallistatin	1.420917988	0.42421859
IMB1_HUMAN	KPNB1	Importin subunit beta‐1	1.847017541	0.36143739

**TABLE 3:** AD vs CTRL: proteins with decreased abundance.

Proteins with decreased abundance				
Entry name	Gene name	Protein name	Log10 *p*‐value	Log2(FC)
OIT3_HUMAN	OIT3	Oncoprotein‐induced transcript 3 protein	1.930804513	−2.5579022
PCBP1_HUMAN	PCBP1	Poly(rC)‐binding protein 1	1.452501853	−2.529085296
ARK72_HUMAN	AKR7A2	Aflatoxin B1 aldehyde reductase member 2	2.102366335	−2.519435474
PDLI7_HUMAN	PDLIM7	PDZ and LIM domain protein 7	1.503722891	−2.350127901
CAVN2_HUMAN	CAVIN2	Caveolae‐associated protein 2	1.45862263	−2.296030317
ZYX_HUMAN	ZYX	Zyxin	1.328376241	−2.265498843
QCR1_HUMAN	UQCRC1	Cytochrome b‐c1 complex subunit 1. mitochondrial	1.501160906	−2.264750344
UBP5_HUMAN	USP5	Ubiquitin carboxyl‐terminal hydrolase 5	1.928493954	−2.106007372
TLN1_HUMAN	TLN1	Talin‐1	1.537018769	−2.082596506
DBNL_HUMAN	DBNL	Drebrin‐like protein	1.416030722	−2.081208502
COR1A_HUMAN	CORO1A	Coronin‐1A	1.617583302	−2.066999708
ALDOA_HUMAN	ALDOA	Fructose‐bisphosphate aldolase A	1.680815075	−2.048437119
PLXB2_HUMAN	PLXNB2	Plexin‐B2	1.75778684	−1.987599237
COR1B_HUMAN	CORO1B	Coronin‐1B	1.399400849	−1.987313952
1433Z_HUMAN	YWHAZ	14‐3‐3 protein zeta/delta	1.387336641	−1.974947793
RSU1_HUMAN	RSU1	Ras suppressor protein 1	1.377309374	−1.847135135
WASP_HUMAN	WAS	Wiskott‐Aldrich syndrome protein	1.565950403	−1.781939234
MTSS1_HUMAN	MTSS1	Protein MTSS 1	1.770617279	−1.685571534
ILK_HUMAN	ILK	Integrin‐linked protein kinase	1.338858144	−1.669870513
PEPD_HUMAN	PEPD	Xaa‐Pro dipeptidase	1.557710901	−1.626527786
VTDB_HUMAN	GC	Vitamin D‐binding protein	1.699345967	−1.521859578
COF1_HUMAN	CFL1	Cofilin‐1	1.740959704	−1.460588591
TBCB_HUMAN	TBCB	Tubulin‐folding cofactor B	1.341693649	−1.3918487
VINC_HUMAN	VCL	Vinculin	1.424933334	−1.389272009
BLVRB_HUMAN	BLVRB	Flavin reductase (NADPH)	1.52174897	−1.313137872
LASP1_HUMAN	LASP1	LIM and SH3 domain protein 1	1.316985004	−1.248676845
FINC_HUMAN	FN1	Fibronectin	1.969991275	−1.238673619
VTNC_HUMAN	VTN	Vitronectin	2.182122267	−1.231091636
CAP1_HUMAN	CAP1	Adenylyl cyclase‐associated protein 1	1.314363192	−1.215120724
TERA_HUMAN	VCP	Transitional endoplasmic reticulum ATPase	1.641027306	−1.213278634
TCPQ_HUMAN	CCT8	T‐complex protein 1 subunit theta	1.422180337	−1.193658011
GSTO1_HUMAN	GSTO1	Glutathione S‐transferase omega‐1	1.791999803	−1.131239482
ANXA3_HUMAN	ANXA3	Annexin A3	1.574144385	−1.120178359
CD5L_HUMAN	CD5L	CD5 antigen‐like	1.315720543	−1.033002853
IGJ_HUMAN	JCHAIN	Immunoglobulin J chain	1.359723652	−0.909084729
SAA4_HUMAN	SAA4	Serum amyloid A‐4 protein	1.68656649	−0.837461063
CO3_HUMAN	C3	Complement C3	1.786976917	−0.722804206
A2AP_HUMAN	SERPINF2	Alpha‐2‐antiplasmin	1.37758094	−0.639695576
HNRPK_HUMAN	HNRNPK	Heterogeneous nuclear ribonucleoprotein K	1.342435942	−0.498254776
ENOA_HUMAN	ENO1	Alpha‐enolase	1.406542821	−0.398424285
KPYM_HUMAN	PKM	Pyruvate kinase PKM	1.437698981	−0.353970528

This quantitative analysis shows a general increased abundance in the AD group of proteins associated with complement systems, components of the ribosomal machinery and small regulatory proteins, such as RPS16, RPS4X, RPL24, RPLP2, RPL12, RPS25, ARF4, RPL23, and RPL18.

Complement factor D (CFD) is consistent with recent longitudinal evidence showing that plasma CFD increases progressively before clinical AD onset and distinguishes AD from other dementia subtypes, implicating the alternative complement pathway as both an early systemic marker and a candidate therapeutic target in preclinical AD [47].

The significant increase of these ribosomal complex proteins in the AD‐derived BC aligns with current literature indicating dysregulation of translational machinery in AD. Previous studies have reported the increased abundance of multiple ribosomal components, including RPLP0, RPSA, and several members of the RPL and RPS families, in brain capillaries isolated from AD patients [48, 49]. Similarly, Fan et al. demonstrated increased abundance of ribosomal proteins such as RPL4, RPL18, RPL18A, and RPS7 and RPS28 in pericytes from AD brains, highlighting that this phenomenon is not restricted to a single vascular cell type but may represent a broader feature of the neurovascular unit in AD [50].

Altered ribosomal protein expression may indicate a cellular stress response and impaired proteostasis in neurodegenerative settings. In the cerebrovascular compartment, increased ribosomal activity could be associated with inflammatory processes, oxidative stress, and blood‐brain barrier dysfunction typical of AD. Importantly, the involvement of ribosomal proteins in extra‐translational functions (e.g., cell cycle control, apoptosis, immune regulation) suggests that their high abundance may actively contribute to disease‐related signaling pathways [49]. Consistent with this view, dysregulated protein abundance has emerged as a shared molecular feature across several neurological disorders [48, 49]. Taken together, our findings reinforce the emerging view that ribosomal protein high abundance is a key feature of AD pathology and extend this concept to BC‐derived samples.

The proteins identified as less abundant in the AD‐derived BC include cytoskeletal regulators, metabolic enzymes, and vascular integrity factors, suggesting a systemic reflection of the neurodegenerative process.

In our study, the most prominent cluster of less abundant proteins belongs to the focal adhesion and actin‐regulatory machinery, including Vinculin, Talin‐1, Zyxin, Cofilin‐1, Coronin‐1, and Drebrin‐like protein. These proteins are essential for maintaining cytoskeletal organization, cell‐matrix interactions, and mechano‐transduction in vascular cells.

Cytoskeletal dynamics play a critical role in AD progression, as they are tightly linked to synaptic structure and function. Disruption of actin remodeling and focal adhesion signaling can impair cellular stability and communication, thereby contributing to synaptic dysfunction and loss, key pathological features that drive neurodegeneration in AD [51, 52, 53, 54]. Indeed, in the AD brain, synaptic plasticity deficits are preceded by the formation of *cofilin‐actin rods* and by the depletion of Drebrin from dendritic spines [53, 54]. The reduced abundance of these cytoskeletal regulators in plasma‐derived BCs may therefore reflect a broader impairment of structural integrity in brain cells, extending beyond neurons and mirroring the systemic nature of AD‐related cytoskeletal dysfunction.

The differential expression analysis of normalized protein abundance also identified a limited presence of proteins involved in the glycolytic metabolism, in particular of alpha‐enolase (ENO1), pyruvate kinase (PKM), and fructose‐bisphosphate aldolase A (ALDOA), alongside the mitochondrial component cytochrome b‐c1 complex subunit 1 (UQCRC1) (Table 3) [55, 56, 57]. The reduced recruitment of these proteins onto the NPs surface suggests a peripheral reflection of the systemic bioenergetic collapse that defines the AD phenotype. This outcome is completely in line with literature where the glycolytic metabolism deficit is reported as a key aspect characterizing the early‐stage AD [58]. Particularly, a reduced glycolytic flux in the brain has been directly correlated with the AD severity showing an increase of plaques and tangles found in the brains [58, 59, 60]. Furthermore, the altered levels of the proteins involved in the glycolytic pathway lead biosynthetic and bioenergetic disorders with abnormal protein deposition and cognitive decline [58, 59, 61]. In our study, the concomitant reduction of glycolytic enzymes and mitochondrial respiratory components points to a multi‐level impairment of cellular energy production. In this context, ALDOA is of particular interest, as it functionally links metabolic pathways to cytoskeletal organization. Its decreased abundance, together with the loss of structural proteins identified here, supports the hypothesis of a coordinated disruption of energy metabolism and cellular architecture. Indeed, ALDOA is a glycolytic enzyme and, by the reversible binding to the actin‐containing filament of cytoskeleton, it can also regulate the cell contraction [55, 62, 63]. Overall, these outcomes indicate that the BC‐based nanosensor can reflect disease‐associated alterations in protein regulation, characterized by increased ribosomal signatures and reduced metabolic and cytoskeletal components in AD samples. Beyond its diagnostic value, the BC signature identified here is also informative about how the Si‐NPs interact with the AD plasma proteome at the molecular level. NPs surface chemistry is well established to shape the BC composition, highlighting the complement components as the protein classes most consistently represented in the BC due to the opsonization process [12, 22, 23].This is consistent with the marked enrichment of CFD in the AD‐derived BC, alongside immunoglobulin‐ and growth‐factor‐binding‐protein components also identified in our dataset (Table 2), and supports the interpretation that the dual Si‐NH_3_^+^/Si‐SO_3_^−^ cocktail efficiently captures complement‐pathway proteins from plasma.

Lipids are the principal component of brain tissue, accounting for approximately 78% of the dry weight of the axonal myelin sheath and 35–40% of neuronal gray matter [64]. Over a century of biochemical research has established that the human brain lipidome is characterized by a high degree of complexity and regional specificity. Key components include phospholipids (primarily phosphatidylcholines and phosphatidylethanolamines), sphingolipids (such as ceramides), and sterols (cholesterol), which exhibit distinct profiles across different brain structures [64, 65]. Myelin membranes are enriched with sphingolipids and cholesterol compared to other cellular components, while synaptic membranes possess unique fatty acid signatures essential for neurotransmission [64]. Recent advancements in MS have enabled compound‐level resolution of these lipids, revealing precise variations across brain regions and cell types. Crucially, systemic alterations in lipid balance have been fundamentally linked to the pathogenesis of various neurodevelopmental and neurodegenerative conditions, including AD [64, 66, 67, 68, 69, 70, 71]. Although lipidomic profiling of the NP‐BC has been investigated across various material and conditions [72, 73, 74], to our knowledge no studies investigated disease‑specific lipid alterations in the corona of NPs exposed to plasma from AD patients. Previous work has focused on general characterization of corona lipids, but not on pathological stratification, underscoring the novelty of the present BC‐associated lipidomic analysis in an AD context. Therefore, to provide a complete view of the systemic changes associated with AD, we analyzed the lipidomic profiles of BCs derived from our Si‐NPs‐based diagnostic platform.

**FIGURE 6:**
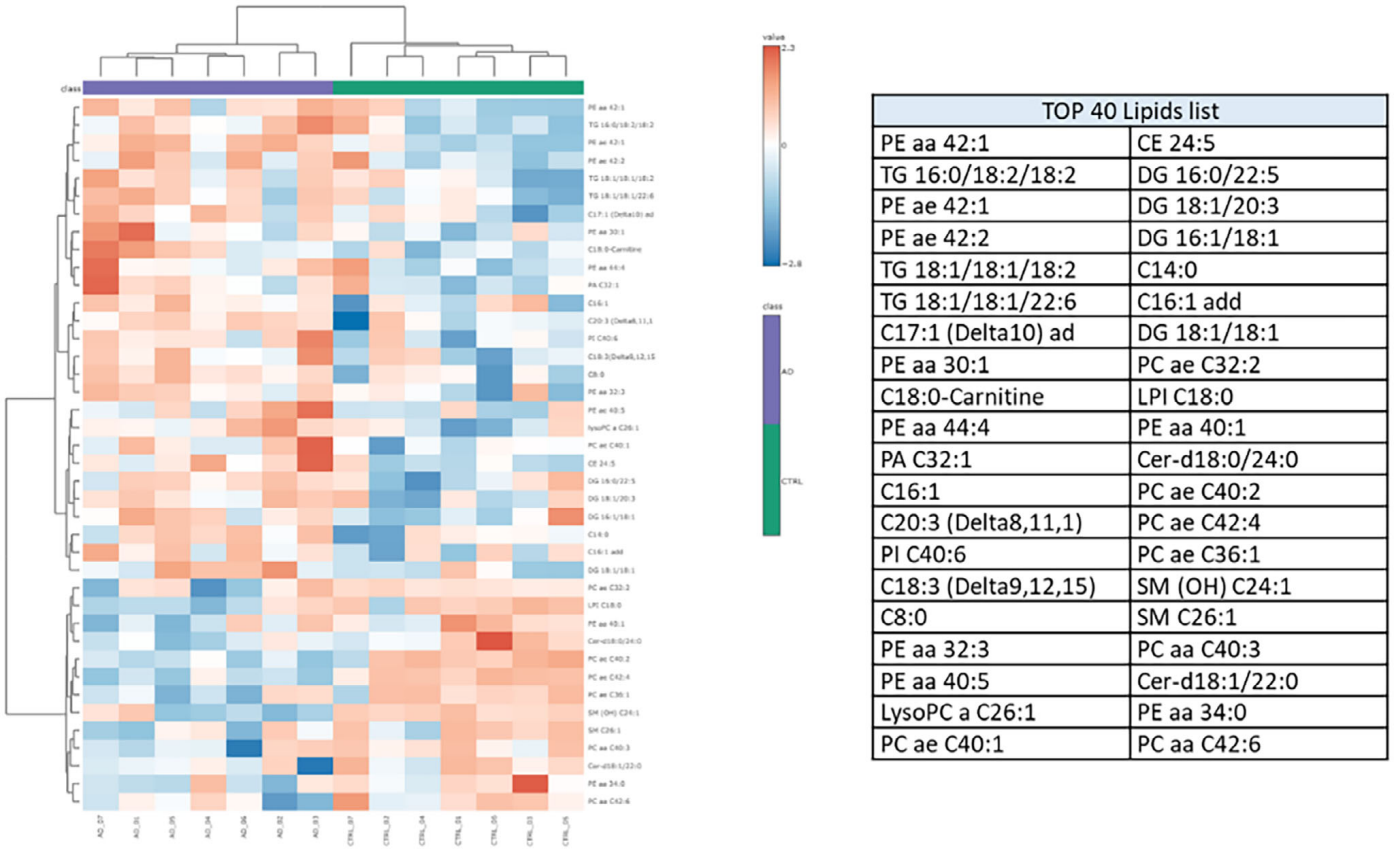
Lipidomic analysis. Heatmap (left) showing the distribution and relative intensity of significantly altered lipids in AD and CTRL groups and table (right) of the top 40 lipids detected during the analysis of BC through UHPLC.

An unsupervised hierarchical clustering heat map was used to visualize differences in lipid abundance (Figure 6). The heatmap, focused on the 40 most variable lipids (table Figure 6), reveals differences in abundance that likely reflect systemic metabolic alterations associated with AD, including changes in membrane composition, signaling lipids, and energy‐related lipid species [75, 76, 77, 78, 79].

**FIGURE 7:**
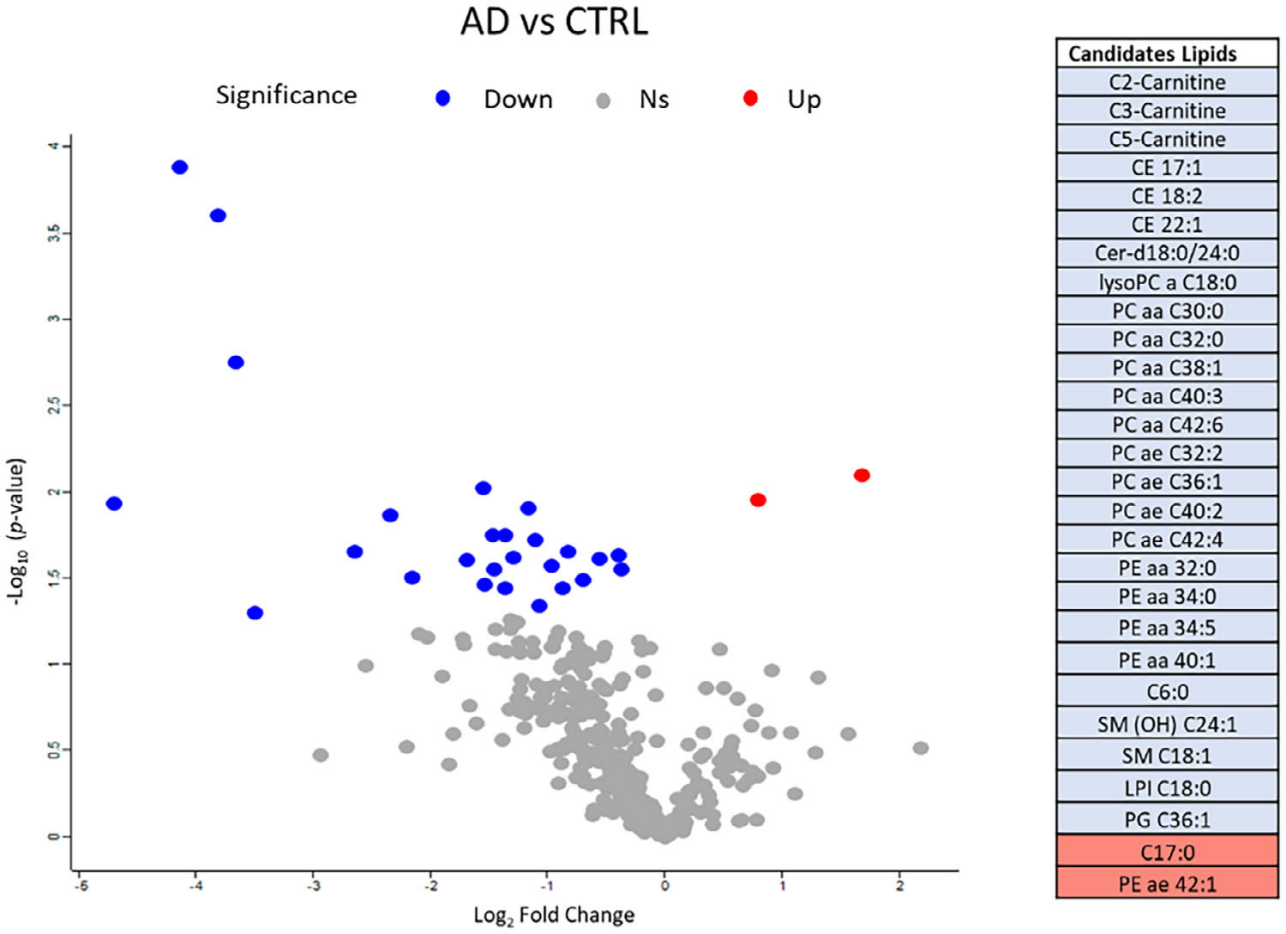
Differential abundance of lipids in AD versus CTRL BC. The volcano plot displays the distribution of lipid species based on their statistical significance ‐Log_10_ (*p*‐value) and magnitude of change (Log_2_ Fold Change). Blue dots represent significantly less abundant lipids in the AD‐derived BC, while red dots indicate high abundant species. Gray dots (Ns) represent lipids that did not meet the significance threshold (*p* > 0.05). The table (on the right) lists candidate lipids, highlighting a predominant depletion of acyl‐carnitines, phosphatidylcholines, and phosphatidylethanolamines in the AD cohort. Only heptadecanoic acid (C17:0) and PE ae 42:1 (highlighted in red) show significant enrichment.

In parallel, univariate statistical analysis was performed and summarized using a volcano plot, enabling the identification of lipid showing both statistically significant and biologically relevant changes between CTRL and AD samples (Figure 7). The analysis highlights a predominant reduction in protein abundance in the AD‐derived BC (blue dots), as further detailed in the accompanying table (Figure 7, right). Specifically, the AD BC shows marked decreases across multiple lipid classes, including acyl‐carnitines, phosphatidylcholines, phosphatidylethanolamines, and sphingomyelins. Notably, short‐chain acyl‐carnitines such as C2, C3, and C5 are significantly reduced, consistent with mitochondrial dysfunction and impaired energy metabolism [80, 81, 82]. In line with our BC‐based proteomic observations, these findings suggest a coordinated reduction of glycolytic and mitochondrial pathways, confirming that energy metabolism represents a critical biochemical aspect of AD progression [83, 84, 85].

In contrast, certain lipids, including heptadecanoic acid (C17:0) and the phosphatidylethanolamine PE ae 42:1, are significantly enriched in the AD‐derived BC. Dysregulation of phosphatidylethanolamines, in particular, has been linked to the main metabolic defects observed in the AD brain, highlighting their potential relevance as disease‐specific lipid markers [86, 87].

Together, these results demonstrate that the BC formed on our Si‐NPs selectively captures molecular features associated with AD, providing insight into both systemic metabolic alterations and potential lipid biomarkers for disease detection and monitoring. This integrated lipidomic fingerprint, combined with proteomic data, reinforces the potential of BC‐based nanosensors as a minimally invasive platform for AD diagnostics and for probing mechanistic aspects of disease progression. From a clinical perspective, these findings support the translational potential of NP‐BC profiling as a minimally invasive approach for AD patient stratification and monitoring. The ability of Si‐NP‐derived BCs to selectively enrich disease‐associated molecular signatures suggests that this platform could complement existing diagnostic tools by providing accessible blood‐based biomarkers reflective of underlying pathological processes. Moreover, the identified panel of BC‐enriched proteins and lipids may enable longitudinal assessment of disease progression and treatment response, highlighting the broader potential of NP‐BC‐based technologies for precision medicine applications in AD [17].

## 4 Conclusion

In the present study, we demonstrate the efficacy of our diagnostic nanotechnological platform, based on BC analysis, for the accurate detection of AD. By leveraging a dual Si‐NPs system integrated with proteomic and lipidomic analyses, we identified distinctive pathological signatures associated with AD. A key strength of our approach is the discovery of a dual molecular shift characterizing the AD environment: alterations in ribosomal machinery and energy metabolism. MS analysis of the BCs revealed increased abundance of ribosomal proteins in AD patients. Concurrently, we observed significant decreased abundance of glycolytic enzymes and mitochondrial respiratory components, mirrored by reduced levels of short‐chain acyl‐carnitines (C2, C3, C5). This metabolic impairment, together with the loss of structural proteins (e.g., Cofilin‐1, Vinculin) and essential membrane phospholipids, defines a comprehensive molecular fingerprint that reflects the AD phenotype.

Overall, our platform overcomes the limitations of single‐marker assays by capturing a multi‐level molecular signature of disease. This BC‐based diagnostic system not only provides a powerful strategy for AD‐associated profiling but also holds potential as a versatile tool for monitoring disease progression and evaluating the efficacy of emerging therapeutic interventions.

## Conflicts of Interest

The authors declare no conflicts of interest.

## Supporting information




**Supporting File 1**: adhm71441‐sup‐0001‐SuppMat.docx.

**Supporting File 2**: adhm71441‐sup‐0002‐TableS2.xlsx.

## Data Availability

The data that support the findings of this study are available on request from the corresponding author. The data are not publicly available due to privacy or ethical restrictions.

## References

[1] C. Corbo , A. A. Li , H. Poustchi , et al., “Analysis of the Human Plasma Proteome Using Multi‐Nanoparticle Protein Corona for Detection of Alzheimer's Disease,” Advanced Healthcare Materials 10 (2021): 2000948. 10.1002/adhm.202000948 33169521

[2] Q. Zheng and X. Wang , “Alzheimer's Disease: Insights into Pathology, Molecular Mechanisms, and Therapy,” Protein & Cell 16 (2025): 83–120. 38733347 10.1093/procel/pwae026 PMC11786724

[3] A. C. Mmadumbu , F. Saeed , F. Ghaleb , and S. N. Qasem , “Early Detection of Alzheimer's Disease Using Deep Learning Methods,” Alzheimer's and Dementia 21 (2025): 70175, 10.1002/ALZ.70175. PMC12069014 40356024

[4] M. Mian , J. Tahiri , R. Eldin , M. Altabaa , U. Sehar , and P. H. Reddy , “Overlooked Cases of Mild Cognitive Impairment: Implications to Early Alzheimer's Disease,” Ageing Research Reviews 98 (2024): 102335, 10.1016/j.arr.2024.102335. 38744405 PMC11180381

[5] R. Molinaro , C. Boada , G. M. Del Rosal , et al., “Vascular Inflammation: A Novel Access Route for Nanomedicine,” Methodist DeBakey Cardiovascular Journal 12 (2016): 169. 27826372 10.14797/mdcj-12-3-169 PMC5098575

[6] M. R. Sepand , M. Ghavami , S. Zanganeh , et al., “Impact of Plasma Concentration of Transferrin on Targeting Capacity of Nanoparticles,” Nanoscale 12 (2020): 4935–4944. 32051994 10.1039/c9nr08784b

[7] C. Mancino , M. Franke , A. Greco , et al., “RNA Therapies for Musculoskeletal Conditions,” Journal of Controlled Release 377 (2025): 756–766. 39617171 10.1016/j.jconrel.2024.11.057

[8] A. P. Porsteinsson , R. S. Isaacson , S. Knox , M. N. Sabbagh , and I. Rubino , “Diagnosis of Early Alzheimer's Disease: Clinical Practice in 2021,” The Journal of Prevention of Alzheimer's Disease 8 (2021): 371–386. 10.14283/jpad.2021.23 PMC12280795 34101796

[9] Y. Chen , M. Al‐Nusaif , S. Li , et al., “Progress on Early Diagnosing Alzheimer's Disease,” Frontiers of Medicine 18 (2024): 446–464. 38769282 10.1007/s11684-023-1047-1 PMC11391414

[10] Y. Dong , X. Song , X. Wang , S. Wang , and Z. He , “The Early Diagnosis of Alzheimer's Disease: Blood‐Based Panel Biomarker Discovery by Proteomics and Metabolomics,” CNS Neuroscience & Therapeutics 30 (2024): 70060, 10.1111/cns.70060. PMC11581788 39572036

[11] M. Kale , N. Wankhede , R. Pawar , et al., “AI‐Driven Innovations in Alzheimer's Disease: Integrating Early Diagnosis, Personalized Treatment, and Prognostic Modelling,” Ageing Research Reviews 101 (2024): 102497. 39293530 10.1016/j.arr.2024.102497

[12] A. Greco , E. Imperlini , G. Y. Lee , M. Serda , S. A. Tobar Leitão , and C. Corbo , “Study of the Biomolecular Corona of Surface‐Modified Polystyrene and Silica Nanoparticles: Application to Cancer,” Nanoscale 17 (2025): 25254–25265. 41134530 10.1039/d5nr03082j

[13] S. A. Tobar Leitão , G. Sausen , Y. Liu , et al., “A Protein Corona‐Based Diagnostic Tool for Eroded Atherosclerotic Plaques,” Small 21 (2025): 2503915. 40321060 10.1002/smll.202503915 PMC12332804

[14] G. Y. Lee , A. A. Li , I. Moon , et al., “Protein Corona Sensor Array Nanosystem for Detection of Coronary Artery Disease,” Small 20 (2024): 2306168, 10.1002/SMLL.202306168. PMC11573401 37880910

[15] P. Bhatt , A. Kamra , A. Chandra , et al., “Construction of a Minimal Sensor Array Using Fingerprint Protein Corona on Nanostars for Detecting Protein Isoforms and Disease States,” ACS Nano (2026): 949–962, 10.1021/ACSNANO.5C16346. 41258927

[16] H. Guo , B. Jin , Z. Zhu , et al., “Nanoparticle–Protein Corona Boosted Cancer Diagnosis with Proteomic Transfer Learning,” ACS Nano 19 (2025): 23592–23605. 40560746 10.1021/acsnano.5c01197

[17] A. Greco and C. Corbo , “Therapeutic Implications of Biomolecular Corona on Lipid Nanoparticle‐Based Gene Delivery Systems,” Small Science 5 (2025): 2500206. 40917404 10.1002/smsc.202500206 PMC12412465

[18] M. R. Mohammadi , C. Corbo , R. Molinaro , and J. R. T. Lakey , “Biohybrid Nanoparticles to Negotiate with Biological Barriers,” Small 15 (2019): 1902333, 10.1002/SMLL.201902333. 31250985

[19] C. Corbo , R. Molinaro , A. Parodi , N. E. Toledano Furman , F. Salvatore , and E. Tasciotti , “The Impact of Nanoparticle Protein Corona on Cytotoxicity, Immunotoxicity and Target Drug Delivery,” Nanomedicine 11 (2016): 81–100. 26653875 10.2217/nnm.15.188 PMC4910943

[20] C. Corbo , R. Molinaro , M. Tabatabaei , O. C. Farokhzad , and M. Mahmoudi , “Personalized Protein Corona on Nanoparticles and Its Clinical Implications,” Biomaterials Science 5 (2017): 378–387. 28133653 10.1039/c6bm00921b PMC5592724

[21] C. Corbo , R. Molinaro , F. Taraballi , et al., “Unveiling the in Vivo Protein Corona of Circulating Leukocyte‐Like Carriers,” ACS Nano 11 (2017): 3262–3273. 28264157 10.1021/acsnano.7b00376

[22] A. Greco and C. Corbo , “Could Selection of Biomolecular Corona Constituents through Nanoparticle Design Produce More Effective Localized Therapies?,” Nanomedicine 20 (2025): 1219–1222. 40088006 10.1080/17435889.2025.2480048 PMC12140490

[23] N. Kamaly , O. C. Farokhzad , and C. Corbo , “Nanoparticle Protein Corona Evolution: from Biological Impact to Biomarker Discovery,” Nanoscale 14 (2022): 1606–1620. 35076049 10.1039/d1nr06580g

[24] M. Hadjidemetriou , J. Rivers‐Auty , L. Papafilippou , et al., “Nanoparticle‐Enabled Enrichment of Longitudinal Blood Proteomic Fingerprints in Alzheimer's Disease,” ACS Nano 15 (2021): 7357–7369. 33730479 10.1021/acsnano.1c00658 PMC8155389

[25] L. Papafilippou , A. Claxton , P. Dark , K. Kostarelos , and M. Hadjidemetriou , “Protein Corona Fingerprinting to Differentiate Sepsis from Non‐Infectious Systemic Inflammation,” Nanoscale 12 (2020): 10240–10253. 32356537 10.1039/d0nr02788j

[26] S. Sheibani , K. Basu , A. Farnudi , et al., “Nanoscale Characterization of the Biomolecular Corona by Cryo‐Electron Microscopy, Cryo‐Electron Tomography, and Image Simulation,” Nature Communications 12 (2021): 573, 10.1038/S41467-020-20884-9. PMC7835367 33495475

[27] W. WU , Q. WU , Q. LIU , et al., “Identification and Characterization of Soft Protein Corona Absorbed on Iron Oxide Nanoparticles,” Chinese Journal of Analytical Chemistry 51 (2023): 100246.

[28] F. Patwekar , M. Patwekar , S. W. Lee , R. Sharma , R. Varghese , and A. Mohammed , “Antibody‐Based Nanoparticles in Alzheimer's Disease: Innovations in Diagnosis and Therapy,” Pathology—Research and Practice 281 (2026): 156421. 41762606 10.1016/j.prp.2026.156421

[29] A. A. Ashkarran , H. Gharibi , S. A. Sadeghi , et al., “Small Molecule Modulation of Protein Corona for Deep Plasma Proteome Profiling,” Nature Communications 15 (2024): 9638. 10.1038/s41467-024-53966-z PMC11544298 39511193

[30] C. Liu , Y. Han , Z. Wang , L. Zhang , and W. Yang , “Preparation of (3‐Aminopropyl)Triethoxysilane‐Modified Silica Particles with Tunable Isoelectric Point,” Langmuir 40 (2024): 12565–12572. 38836786 10.1021/acs.langmuir.4c01027

[31] G. Bashiri , M. S. Padilla , K. L. Swingle , S. J. Shepherd , M. J. Mitchell , and K. Wang , “Nanoparticle Protein Corona: from Structure and Function to Therapeutic Targeting,” Lab on a Chip 23 (2023): 1432–1466. 36655824 10.1039/d2lc00799a PMC10013352

[32] M. P. Vincent , S. Bobbala , N. B. Karabin , et al., “Surface Chemistry‐Mediated Modulation of Adsorbed Albumin Folding state Specifies Nanocarrier Clearance by Distinct Macrophage Subsets,” Nature Communications 12 (2021): 648. 10.1038/s41467-020-20886-7 PMC7844416 33510170

[33] D. Prozeller , C. Rosenauer , S. Morsbach , and K. Landfester , “Immunoglobulins on the Surface of Differently Charged Polymer Nanoparticles,” Biointerphases 15 (2020): 31009. 10.1116/6.0000139 32486649

[34] H. Lee , “Effects of Nanoparticle Electrostatics and Protein–Protein Interactions on Corona Formation: Conformation and Hydrodynamics,” Small 16 (2020): 1906598. 10.1002/smll.201906598 32022403

[35] W. Stöber , A. Fink , and E. Bohn , “Controlled Growth of Monodisperse Silica Spheres in the Micron Size Range,” Journal of Colloid & Interface Science 26 (1968): 62–69.

[36] C. P. Tsai , C. Y. Chen , Y. Hung , F. H. Chang , and C. Y. Mou , “Monoclonal Antibody‐Functionalized Mesoporous Silica Nanoparticles (MSN) for Selective Targeting Breast Cancer Cells,” Journal of Materials Chemistry 19 (2009): 5737.

[37] F. Kunc , V. Balhara , A. Brinkmann , Y. Sun , D. M. Leek , and L. J. Johnston , “Quantification and Stability Determination of Surface Amine Groups on Silica Nanoparticles Using Solution NMR,” Analytical Chemistry 90 (2018): 13322–13330. 30372033 10.1021/acs.analchem.8b02803

[38] N. Rasool , D. Negi , and Y. Singh , “Thiol‐Functionalized, Antioxidant, and Osteogenic Mesoporous Silica Nanoparticles for Osteoporosis,” ACS Biomaterials Science & Engineering 9 (2023): 3535–3545. 37172017 10.1021/acsbiomaterials.3c00479

[39] C. S. Hughes , S. Moggridge , T. Müller , P. H. Sorensen , G. B. Morin , and J. Krijgsveld , “Single‐Pot, Solid‐Phase‐Enhanced Sample Preparation for Proteomics Experiments,” Nature Protocols 14 (2018): 68–85. 10.1038/s41596-018-0082-x 30464214

[40] S. Tyanova , T. Temu , P. Sinitcyn , et al., “The Perseus Computational Platform for Comprehensive Analysis of (prote)Omics Data,” Nature Methods 13 (2016): 731–740. 27348712 10.1038/nmeth.3901

[41] Z. Pang , Y. Lu , G. Zhou , et al., “MetaboAnalyst 6.0: towards a Unified Platform for Metabolomics Data Processing, Analysis and Interpretation,” Nucleic Acids Research 52 (2024): W398–W406. 38587201 10.1093/nar/gkae253 PMC11223798

[42] D. W. Huang , B. T. Sherman , and R. A. Lempicki , “Systematic and Integrative Analysis of Large Gene Lists Using DAVID Bioinformatics Resources,” Nature Protocols 4 (2008): 44–57. 10.1038/nprot.2008.211 19131956

[43] B. T. Sherman , M. Hao , J. Qiu , et al., “DAVID: a Web Server for Functional Enrichment Analysis and Functional Annotation of Gene Lists (2021 update),” Nucleic Acids Research 50 (2022): W216–W221. 35325185 10.1093/nar/gkac194 PMC9252805

[44] I. A. Rahman , P. Vejayakumaran , C. S. Sipaut , J. Ismail , and C. K. Chee , “Size‐Dependent Physicochemical and Optical Properties of Silica Nanoparticles,” Materials Chemistry and Physics 114 (2009): 328–332.

[45] M. Kokkinopoulou , J. Simon , K. Landfester , V. Mailänder , and I. Lieberwirth , “Visualization of the Protein Corona: towards a Biomolecular Understanding of Nanoparticle‐Cell‐Interactions,” Nanoscale 9 (2017): 8858–8870. 28632260 10.1039/c7nr02977b

[46] S. Palchetti , V. Colapicchioni , L. Digiacomo , et al., “The Protein Corona of Circulating PEGylated Liposomes,” Biochimica et Biophysica Acta (BBA)—Biomembranes 1858 (2016): 189–196. 26607013 10.1016/j.bbamem.2015.11.012

[47] X. Fu , H. Cai , S. Quan , et al., “Systemic Complement Factors in Aging, Alzheimer's Disease and Other Dementias: A Longitudinal Study over 10 Years,” Molecular Neurodegeneration 21 (2026): 11. 41526986 10.1186/s13024-026-00927-3 PMC12888596

[48] M. Suzuki , K. Tezuka , T. Handa , et al., “Upregulation of Ribosome Complexes at the Blood‐Brain Barrier in Alzheimer's disease Patients,” Journal of Cerebral Blood Flow & Metabolism 42 (2022): 2134–2150. 35766008 10.1177/0271678X221111602 PMC9580172

[49] A. Yamakawa , M. Suganuma , R. Mitsumori , S. Niida , K. Ozaki , and D. Shigemizu , “Alzheimer's Disease May Develop from Changes in the Immune System, Cell Cycle, and Protein Processing Following Alterations in Ribosome Function,” Scientific Reports 15 (2025): 3838. 39885278 10.1038/s41598-025-88526-y PMC11782650

[50] L. Y. Fan , J. Yang , R. Y. Liu , Y. Kong , G. Y. Guo , and Y. M. Xu , “Integrating Single‐Nucleus Sequence Profiling to Reveal the Transcriptional Dynamics of Alzheimer's Disease, Parkinson's Disease, and Multiple Sclerosis,” Journal of Translational Medicine 21 (2023): 649. 37735671 10.1186/s12967-023-04516-6 PMC10515258

[51] S. Pelucchi , R. Stringhi , and E. Marcello , “Dendritic Spines in Alzheimer's Disease: How the Actin Cytoskeleton Contributes to Synaptic Failure,” International Journal of Molecular Sciences 21 (2020): 908. 32019166 10.3390/ijms21030908 PMC7036943

[52] G. Jiang , G. Xie , X. Li , et al., “Cytoskeletal Proteins and Alzheimer's Disease Pathogenesis: Focusing on the Interplay with Tau Pathology,” Biomolecules 15 (2025): 831, 10.3390/BIOM15060831. 40563471 PMC12190275

[53] Q. Wang , W. Yuan , X. Yang , Y. Wang , Y. Li , and H. Qiao , “Role of Cofilin in Alzheimer's Disease,” Frontiers in Cell and Developmental Biology 8 (2020): 584898. 33324642 10.3389/fcell.2020.584898 PMC7726191

[54] L. Zhao , Q. L. Ma , F. Calon , et al., “Role of p21‐Activated Kinase Pathway Defects in the Cognitive Deficits of Alzheimer Disease,” Nature Neuroscience 9 (2006): 234–242. 16415866 10.1038/nn1630

[55] L. M. Nascentes Melo , F. Cansiz , and A. Tasdogan , “Aldolase A: The Broker of Glycolysis,” Nature Metabolism 7 (2025): 242–244. 10.1038/s42255-024-01202-9 39833611

[56] C. K. Huang , Y. Sun , L. Lv , and Y. Ping , “ENO1 and Cancer,” Molecular Therapy—Oncolytics 24 (2022): 288–298. 35434271 10.1016/j.omto.2021.12.026 PMC8987341

[57] A. Nieborak , S. Lukauskas , J. Capellades , et al., “Depletion of Pyruvate Kinase (PK) Activity Causes Glycolytic Intermediate Imbalances and Reveals a PK‐TXNIP Regulatory Axis,” Molecular Metabolism 74 (2023): 101748. 37290673 10.1016/j.molmet.2023.101748 PMC10336528

[58] X. Zhang , N. Alshakhshir , and L. Zhao , “Glycolytic Metabolism, Brain Resilience, and Alzheimer's Disease,” Frontiers in Neuroscience 15 (2021): 662242, 10.3389/FNINS.2021.662242. 33994936 PMC8113697

[59] S. M. Bell , H. Wareing , F. Capriglia , et al., “Increasing Hexokinase 1 Expression Improves Mitochondrial and Glycolytic Functional Deficits Seen in Sporadic Alzheimer's Disease Astrocytes,” Molecular Psychiatry 30 (2024): 1369–1382. 39271753 10.1038/s41380-024-02746-8 PMC11919762

[60] Y. An , V. R. Varma , S. Varma , et al., “Evidence for Brain Glucose Dysregulation in Alzheimer's Disease,” Alzheimer's & Dementia 14 (2018): 318–329. 10.1016/j.jalz.2017.09.011 PMC5866736 29055815

[61] L. Wu , X. Zhang , and L. Zhao , “Human ApoE Isoforms Differentially Modulate Brain Glucose and Ketone Body Metabolism: Implications for Alzheimer's Disease Risk Reduction and Early Intervention,” The Journal of Neuroscience 38 (2018): 6665–6681. 29967007 10.1523/JNEUROSCI.2262-17.2018 PMC6067075

[62] B. R. Berridge , J. F. Van Vleet , and E. Herman , Haschek and Rousseaux's Handbook of Toxicologic Pathology, 3rd edn., Vol. 1–3 (Elsevier, 2013): 1567.

[63] A. Gizak , J. Wiśniewski , P. Heron , P. Mamczur , J. Sygusch , and D. Rakus , “Targeting a Moonlighting Function of Aldolase Induces Apoptosis in Cancer Cells,” Cell Death & Disease 10 (2019): 712. 31558701 10.1038/s41419-019-1968-4 PMC6763475

[64] M. Osetrova , A. Tkachev , W. Mair , et al., “Lipidome Atlas of the Adult human Brain,” Nature Communications 15 (2024): 4455. 10.1038/s41467-024-48734-y PMC11127996 38796479

[65] J. H. Yoon , Y. Seo , Y. S. Jo , et al., “Brain Lipidomics: from Functional Landscape to Clinical Significance,” Science Advances 8 (2022): adc9317. 10.1126/sciadv.adc9317 PMC9481132 36112688

[66] R. B. Chan , T. G. Oliveira , E. P. Cortes , et al., “Comparative Lipidomic Analysis of Mouse and Human Brain with Alzheimer Disease,” Journal of Biological Chemistry 287 (2012): 2678–2688. 22134919 10.1074/jbc.M111.274142 PMC3268426

[67] M. Jové , N. Mota‐Martorell , P. Torres , M. Portero‐Otin , I. Ferrer , and R. Pamplona , “New Insights into human Prefrontal Cortex Aging with a Lipidomics Perspective,” Expert Review of Proteomics 18 (2021): 333–347. 34098823 10.1080/14789450.2021.1940142

[68] C. C. Smith , D. L. Sheedy , H. P. McEwen , A. S. Don , J. J. Kril , and G. T. Sutherland , “Lipidome Changes in Alcohol‐Related Brain Damage,” Journal of Neurochemistry 160 (2022): 271–282. 34699608 10.1111/jnc.15530

[69] M. G. Krokidis , K. A. Pucha , M. Mustapic , T. P. Exarchos , P. Vlamos , and D. Kapogiannis , “Lipidomic Analysis of Plasma Extracellular Vesicles Derived from Alzheimer's Disease Patients,” Cells 13 (2024): 702. 38667317 10.3390/cells13080702 PMC11049154

[70] M. J. Khan , N. A. Chung , S. Hansen , et al., “Targeted Lipidomics To Measure Phospholipids and Sphingomyelins in Plasma: A Pilot Study To Understand the Impact of Race/Ethnicity in Alzheimer's Disease,” Analytical Chemistry 94 (2022): 4165–4174. 35235294 10.1021/acs.analchem.1c03821 PMC9126486

[71] B. E. Makki and S. Rahman , “Alzheimer's Disease in Diabetic Patients: A Lipidomic Prospect,” Neuroscience 530 (2023): 79–94. 37652288 10.1016/j.neuroscience.2023.08.033

[72] K. Liu , R. Nilsson , E. Lázaro‐Ibáñez , et al., “Multiomics Analysis of Naturally Efficacious Lipid Nanoparticle Coronas Reveals High‐Density Lipoprotein Is Necessary for Their Function,” Nature Communications 14 (2023): 4007. 10.1038/s41467-023-39768-9 PMC10325984 37414857

[73] G. La Barbera , A. L. Capriotti , G. Caracciolo , et al., “A Comprehensive Analysis of Liposomal Biomolecular Corona Upon Human Plasma Incubation: The Evolution towards the Lipid Corona,” Talanta 209 (2020): 120487. 31892008 10.1016/j.talanta.2019.120487

[74] C. Braccia , V. Castagnola , E. Vázquez , et al., “The Lipid Composition of Few Layers Graphene and Graphene Oxide Biomolecular Corona,” Carbon 185 (2021): 591–598.

[75] G. Van Meer , D. R. Voelker , and G. W. Feigenson , “Membrane Lipids: Where They Are and How They Behave,” Nature Reviews Molecular Cell Biology 9 (2008): 112–124. 18216768 10.1038/nrm2330 PMC2642958

[76] M. P. Wymann and R. Schneiter , “Lipid Signalling in Disease,” Nature Reviews Molecular Cell Biology 9 (2008): 162–176. 18216772 10.1038/nrm2335

[77] Y. A. Hannun and L. M. Obeid , “Principles of Bioactive Lipid Signalling: Lessons from Sphingolipids,” Nature Reviews Molecular Cell Biology 9 (2008): 139–150. 18216770 10.1038/nrm2329

[78] N. Longo , M. Frigeni , and M. Pasquali , “Carnitine Transport and Fatty Acid Oxidation,” Biochimica et Biophysica Acta (BBA)—Molecular Cell Research 1863 (2016): 2422–2435. 26828774 10.1016/j.bbamcr.2016.01.023 PMC4967041

[79] X. Han , Lipidomics: Comprehensive Mass Spectrometry of Lipids (Wiley, 2016), 1.

[80] S. Zhao , X. F. Feng , T. Huang , et al., “The Association Between Acylcarnitine Metabolites and Cardiovascular Disease in Chinese Patients With Type 2 Diabetes Mellitus,” Frontiers in Endocrinology (Lausanne) 11 (2020): 481835. 10.3389/fendo.2020.00212 PMC7214635 32431666

[81] F. Xiang , Z. Zhang , J. Xie , et al., “Comprehensive Review of the Expanding Roles of the Carnitine Pool in Metabolic Physiology: beyond Fatty Acid Oxidation,” Journal of Translational Medicine 23 (2025): 324. 40087749 10.1186/s12967-025-06341-5 PMC11907856

[82] M. J. Miller , K. Cusmano‐Ozog , D. Oglesbee , and S. Young , “Laboratory Analysis of Acylcarnitines, 2020 Update: A Technical Standard of the American College of Medical Genetics and Genomics (ACMG),” Genetics in Medicine 23 (2021): 249–258. 33071282 10.1038/s41436-020-00990-1

[83] F. Yin , “Lipid Metabolism and Alzheimer's Disease: Clinical Evidence, Mechanistic Link and Therapeutic Promise,” The FEBS Journal 290 (2023): 1420–1453. 34997690 10.1111/febs.16344 PMC9259766

[84] D. Kapogiannis and M. P. Mattson , “Disrupted Energy Metabolism and Neuronal Circuit Dysfunction in Cognitive Impairment and Alzheimer's Disease,” The Lancet Neurology 10 (2010): 187–198. 21147038 10.1016/S1474-4422(10)70277-5 PMC3026092

[85] J. Fu and L. An , “Histone Methylation, Energy Metabolism, and Alzheimer's Disease,” Aging & Disease 16 (2024): 2831–2858. 39656495 10.14336/AD.2024.0899 PMC12339095

[86] J. K. Blusztajn and B. E. Slack , “Accelerated Breakdown of Phosphatidylcholine and Phosphatidylethanolamine Is a Predominant Brain Metabolic Defect in Alzheimer's Disease,” Journal of Alzheimer's Disease 93 (2023): 1285–1289. 10.3233/JAD-230061 PMC10885637 37182883

[87] J. Korbecki , M. Bosiacki , P. Kupnicka , et al., “Biochemistry and Diseases Related to the Interconversion of Phosphatidylcholine, Phosphatidylethanolamine, and Phosphatidylserine,” International Journal of Molecular Sciences 25 (2024): 10745. 39409074 10.3390/ijms251910745 PMC11477190

