# Supplementary material for: Engineering Nanoplatforms for Alzheimer's Disease Detection via Biomolecular Corona Proteomic and Lipidomic Profiling
Source: Adv Healthc Mater. 2026 Jul 11;15(31):e71441. doi: 10.1002/adhm.71441 (PMC13495923; doi:10.1002/adhm.71441)
Supplement: Supplementary file 1 [file ADHM-15-0-s002.docx]

**Engineering Nanoplatforms for Alzheimer’s Disease Detection via Biomolecular Corona Proteomic and Lipidomic Profiling**

Antonietta Greco^1^, Roberto Baretta^2^, Andrea Di Fonzo^3^, Andrea Armirotti^3^, Stefano Cinti^,4,5,6^, Mara Mirasoli^7^, Marco Frasconi^2^, and Claudia Corbo^1,8^*

**Figure S1.** TEM images of pristine Si NPs (**A**), Si–NH_3_^+^ NPs (**B**) and Si–SO_3_^–^ NPs (**C**). Scale bar 200 nm.

**Table S1.** **NPs DLS analysis**

Characterization of Si-NH_3_^+^ NPs and Si-SO_3_^-^ NPs after incubation in AD and no-AD human plasmas. Results represent the mean ± S.D. of 7 independent batches (n = 7).

| Sample | Size ± S.D. (nm) | PDI ± S.D. | Z-Potential ± S.D. (mV) |
| --- | --- | --- | --- |
| Si-NH_3_^+^ NPs AD | 185.34 ± 3.17 | 0.151 ± 0.021 | -38.42 ± 6.12 |
| Si-NH_3_^+^ NPs CTRL | 179.66 ± 4.23 | 0.174 ± 0.033 | -40.15 ± 3.24 |
| Si-SO_3_^-^ NPs AD | 188.37 ± 6.44 | 0.159 ± 0.010 | -63.61 ± 4.87 |
| Si-SO_3_^-^ NPs CTRL | 192.94 ± 2.57 | 0.163 ± 0.052 | -61.03 ± 2.90 |
